# Using blood methylomes to predict response to amisulpride in the first-episode psychosis in the OPTiMiSE cohort

**DOI:** 10.1038/s41398-025-03561-7

**Published:** 2025-10-06

**Authors:** Ana Lokmer, Réjane Troudet, Delphine Bacq-Daian, Anne Boland-Auge, Caroline Barau, the OPTiMiSE study group, Marion Leboyer, Stéphane Jamain, Jean-François Deleuze, Marion Leboyer, Stéphane Jamain

**Affiliations:** 1 Univ Paris Est Créteil, INSERM, IMRB, Translational Neuropsychiatry, Créteil, France; 2 Fondation FondaMental, Créteil, France; 3 Université Paris-Saclay, CEA, Centre National de Recherche en Génomique Humaine (CNRGH), Evry, France; 4 AP-HP, Hôpitaux Universitaires H. Mondor, Plateforme de Ressources Biologiques, Créteil, France; 5 AP-HP, Hôpitaux Universitaires H. Mondor, DMU IMPACT, Créteil, France

**Keywords:** Personalized medicine, Predictive markers, Schizophrenia

## Abstract

Treatment of schizophrenia relies heavily on the use of antipsychotic drugs. Their efficacy is at present determined by lengthy trial-and-error approach, calling for more efficient strategies based on personalized medicine. Here, we present a prospective study of 116 first-episode-psychosis (FEP) patients from the OPTiMiSE cohort, aiming to identify blood epigenomic biomarkers predicting response to amisulpride and to shed light on involved mechanisms by linking the observed methylation patterns to genetic variation and gene expression. The analysis of 210 paired (baseline and follow-up) blood methylomes revealed 67 regions stably differentially methylated between good and bad responders and 197 regions with response-specific dynamics. The former were primarily enriched in functions related to neurotransmission and synapse assembly, the latter in immunity and inflammation. Baseline methylation values of three of these candidate regions, situated within *HOXA, HTR2A* and *PRR5* genes, were selected as good predictors (10x cross-validated Matthews correlation coefficient = 0.81) of amisulpride response in our cohort. Screening for associations between the methylation of the selected regions and the genetic variants (SNPs) in a 1MBp surroundings revealed a high degree of genetic control for *HTR2A*, but not for *HOXA* or *PRR5* regions. Whereas we detected multiple correlations between methylation and gene expression, few were temporally stable, such as the correlation between *HOXA5* and *SKAP2* expression, a gene affecting susceptibility to schizophrenia. Our findings demonstrate the strengths of prospective design in response-biomarker research and suggest that epigenetic variation associated with antipsychotic response is shaped by both the environmental and genetic factors.

## Introduction

Schizophrenia (SCZ) affects only around 0.3% of the global population [1], but its severity, chronicity and frequent comorbidities result in a disproportionate effect on health and economic systems [2]. Schizophrenia is mainly treated by antipsychotics, whose efficacy is highly variable and unpredictable [3]. Considering the severity of disease symptoms and frequent side effects of antipsychotics [4], substituting current time-consuming switching strategies [5] with a biomarker-based, precision.medicine approach, would benefit both patients and healthcare systems.

Genetic polymorphisms represent a natural first choice in the quest for straightforward and accessible biomarkers. However, none of the variants that have been associated with antipsychotic response and adverse effects, many of which involved in neurotransmission and xenobiotics metabolism, reliably predict treatment response [6]. Similar to the etiology of schizophrenia [7], the treatment response variability seems to arise through an interplay of genetic and environmental factors and probably also mirrors clinical heterogeneity [8]. Gene expression and epigenetic marks reflect all these levels of variation, potentially providing more specific biomarkers than polymorphisms [9]. Although numerous studies examined the link between antipsychotic response and gene expression [10] or epigenetic variation (review in e.g. Burghardt et al. [11]), very few [12–15] utilized a prospective design, the most suitable choice for the predictive-biomarker discovery. Inconsistent findings of these studies can be explained by differences in genomic coverage, methodological choices and variability of the examined populations and antipsychotics. Neither Rukova et al. [12] nor Lokmer et al. [13] succeeded in predicting treatment response from baseline methylation values, although Lokmer et al. [13] were able to predict the response to risperidone from the combined baseline and follow-up methylation of four CpGs in an Indian cohort. Tang et al. [14] focused exclusively on immunity-related genes and reported three CpGs as response biomarkers in Han population, none of which could be associated with gene expression variation.

**Fig. 1:**
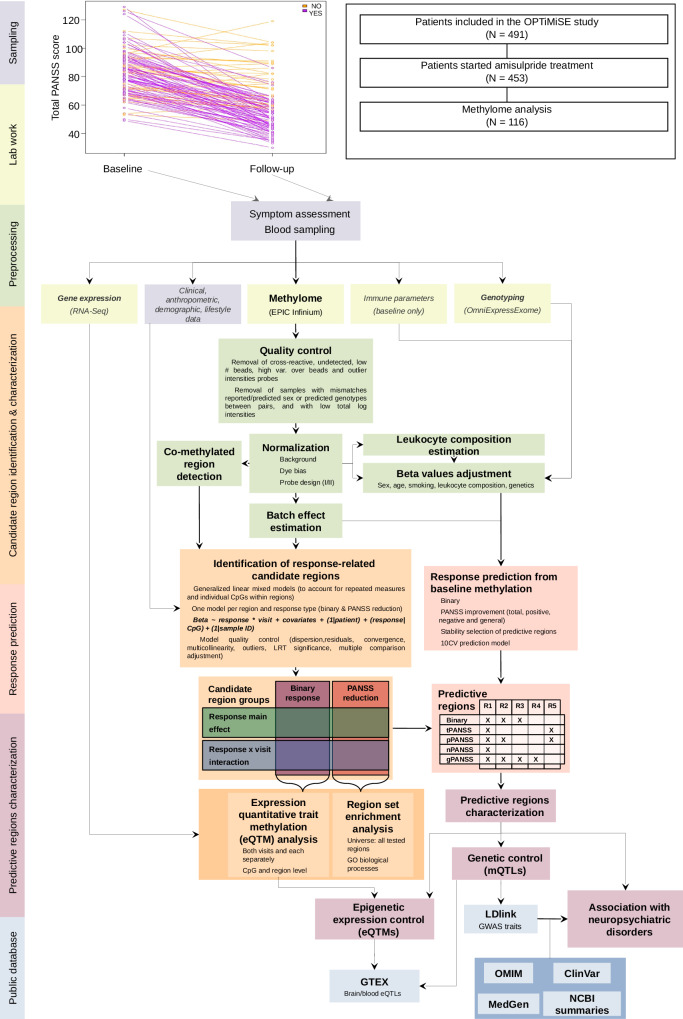
Flowchart of the analysis workflow performed in the study with the first phase of the OPTiMiSE study flowchart in the inset. Response trajectories for individual patients showing total PANSS scores at baseline and follow-up visits are shown on the top of the figure. The rectangles are colored according to study stages or type of data as indicated on the left side of the figure.

Here, we present a prospective, repeated-measures study of blood genome-wide methylation of 116 first-episode psychosis patients from the OPTiMiSE clinical trial [16, 17], aiming, firstly, to predict the response to amisulpride after four weeks of treatment using baseline methylation, and secondly, to identify stable and treatment-induced epigenetic differences between the good and bad responders. In addition, we combined epigenetic, genetic and gene expression data from OPTiMiSE cohort with the knowledge from the public databases to examine how genetic, epigenetic and low-level phenotypic variation interact to shape response to antipsychotic treatment (Fig. 1).

## Methods

### Ethics approval and consent to participate

All methods were performed in accordance with relevant guidelines and regulations. The cohort is a subset of patients from the first phase of the OPTiMiSE (Optimization of Treatment and Management of Schizophrenia in Europe, NCT01248195) clinical trial [16, 17]. A written informed consent was obtained from all participants prior their inclusion in the study. All inclusion sites obtained local ethical approval and this research was approved by an Institutional Review Board.

### Participant characteristics and study design

The cohort comprises 116 adults (41 women and 75 men) from twelve European countries, experiencing first episode psychosis (FEP) for less than two years. Only individuals whose gender identity corresponded with their genetic sex were included. The participants had either been antipsychotic-naive or minimally treated at baseline, when they were prescribed 200–800 mg/day amisulpride orally. Compliance was confirmed by amisulpride concentration blood measurements. Amisulpride was chosen based on its comparatively good efficacy and relatively narrow side-effects profile [18, 19].

Symptom severity was assessed using total Positive and Negative Syndrome Scale (PANSS) score at baseline and four weeks later at follow-up. Patients with at least 20% reduction of PANSS score at follow-up were classified as (good) responders, while those with PANSS reduction below this threshold were considered as bad or non-responders (Fig. 1) [20].

Blood for genetic, epigenetic, gene expression and immune parameter analysis was collected during both visits. Genotyping revealed 104 patients were of European, eight of Asian and four of African ancestry. No further data regarding ethnicity were collected. Cytokine levels were measured due to their potential involvement in antipsychotic response [21]. Additional details regarding genotyping, gene expression and cytokine measurements can be found in [16, 20, 22].

### Generation and quality control of blood methylome data

Laboratory protocols, data generation and quality control procedures have been previously published [13] and detailed methods are also described in Supplementary Materials and Methods. All analyses have been conducted in R statistical framework version 4.1.3 [23]. Briefly, genome-wide methylation of bisulphite-converted blood DNA was assessed using Infinium Methylation EPIC v1.0 BeadChip Illumina Inc., USA) according to the manufacturer’s protocol. Quality control and normalization were conducted following published recommendations [24] and included background, dye- and design-bias normalization, preceded by the exclusion of cross-reactive [25] and undetected (background intensity) probes and low-quality samples (i.e. samples with low median intensity, many undetected probes or genotype of sex mismatch between paired samples).

### Detection and functional characterization of differentially methylated regions

To increase statistical power and reproducibility, we performed a region-based analysis. Regions were identified with the unsupervised method implemented in the *coMethDMR* v.1.2.0 package [26] with default parameters. To identify response-associated regions, we used random coefficient mixed effects beta family models [27], with amisulpride response defined either as categorical variable or as %PANSS reduction. As methylation is measured by beta values, i.e. by the proportion/percentage of methylated sites, we used beta family (a distribution restricted to 0–1 interval) with logit link for modelling. We were interested in both stable methylome differences between the good and bad responders (main effect of response) and in response-specific temporal shifts following antipsychotic treatment (response x visit interaction or response-specific treatment effects). The models included following covariates: age, sex, smoking, alcohol use, body mass index (BMI), duration of untreated psychosis (DUP), leukocyte composition (Supplementary Fig. S1), and covariates accounting for population genetic diversity and batch effects. Patient, sample and probe IDs, included as random effects, accounted for repeated measures design, and for the within-region methylation variability, respectively [26].

We investigated functional enrichment of differentially methylated regions compared to the universe comprising all tested regions [13]. In addition, we checked for any associations with psychiatric, neurodevelopmental or neurodegenerative disorders in the public databases.

We conducted expression quantitative trait methylation (eQTM) analysis by calculating repeated-measures (rmcorr, to account for the CpG-level variability within a region [28]) and Spearman’s rho (for the region mean and individual CpG-level) between methylation and gene expression [20] for all the *cis* region-gene pairs. We did this for both visits together and for each one separately and kept only correlations with the lower 95% confidence interval > 0.1, followed by enrichment analysis.

### Response prediction from differentially methylated regions

We used generalized additive modelling fitted by gradient boosting (*mboost* v. 2.9–5 package [29]) to predict treatment response from the covariate-corrected baseline methylation values of the regions selected as described above. We performed the analysis for dichotomous response and for reduction of total PANSS and its subscores, for all individuals and for the indivduals of European ancestry only. False positive rate was minimized by stability variable selection procedure [30], with 0.75 selection probability cutoff and upper bound for the per-family error-rate PFER = 1. The regions surviving these cutoffs were used to build the final 10x cross-validated predictive models. We used R2 for assessing the performances of PANSS reduction models, and Matthews correlation coefficient (MCC) for dichotomous response, a performance measure suitable for the unbalanced designs [31].

### Detection of methylation quantitative trait loci (mQTLs) associated with the response-predicting regions

To identify methylation quantitative trait loci (mQTLs) in a 1MBp window surrounding the response-predicting regions, we genotyped patients using the OmniExpressExome 8v1-4 A1 BeadChip (Illumina Inc., USA) and performed quality control and imputation following published procedures [32]. We analyzed a single representative from each group of perfectly collinear SNPs (linkage R^2^ = 1) by linear modelling, with genotype as an explanatory variable and covariate-corrected methylation value as a dependent variable. We ran models for both the mean methylation value of a region and for each CpG in the region separately. We considered only SNPs with Benjamini-Hochberg-adjusted p-values < 0.05 and used R-squared as a measure of effect size. We queried public databases using the LDlink tools [33] to search for published associations between our mQTLs and psychiatric diseases and nervous system functioning in “EUR” superpopulation, as 90% participants were of European origin. We then used Fisher’s exact test to find GWAS traits over-represented in our mQTL set as compared to those associated with any SNP in the queried 1MBp window.

## Results

### Good response correlates with leukocyte composition and immunological factors

We observed no differences in sex ratio, BMI, substance abuse or baseline PANSS score between the good (*N* = 83, 72%) and bad (*N* = 33, 28%) responders (Supplementary Table S1). However, good responders were older (t = −2.09, df = 63. 82, *p* = 0.041, effect size = 0. 25) and had shorter duration of untreated psychosis (DUP, U = 2.26, df = 49.10, *p* = 0.024, effect size = 0.21). They also had a higher blood concentration of the CCL22 chemokine (t = −2.65, df = 70.52, *p* = 0.01, effect size = 0.30) and a lower NK-cell relative abundance (t = 2.561 df = 48.10, *p* = 0.014, effect size = 0.35) at baseline. Amisulpride dose and blood concentration tended towards lower values in good responders (dose: t = 1.874 df = 43.21, *p* = 0.068; concentration: t = 1.892 df = 43.77, *p* = 0.065). Furthermore, we detected a weak genetic effect (genetic PC1, distinguishing individuals of African ancestry from the rest, t = 1.98, df =113.52, *p* = 0.05, effect size = 0.18). Finally, the distribution of good and bad responders varied across collection centres (Fisher’s exact test, *p* = 0.021), largely due to the absence of good responders in the centre 17 in Spain.

### Amisulpride response is associated with methylation in regions involved in development, neurotransmission, inflammation and other signaling and immunity-related processes

Multiple-comparison-corrected analysis of 210 paired samples that passed quality control revealed 67 regions with average methylation differences between good and bad responders (main effect of response, Supplementary Table S2) and 197 regions with response-specific treatment effect (visit x response interaction, Supplementary Table S3), with three regions found in both groups (Supplementary Fig. S2). The regions associated with a positive response defined as PANSS reduction, were mostly distinct from those significant for the dichotomous response (Supplementary Table S4, Supplementary Table S5, Supplementary Fig. S2). However, since the magnitude of coefficient estimates from dichotomous and continuous models were strongly correlated (response main effect: r [95%CI] = 0.84 [0.83, 0.85], visit x response interaction: r [95%CI] = 0.86 [0.85, 0.87]) when disregarding the statistical significance, we assumed that the differences arose due to technical rather than to biological reasons and we therefore focus primarily on the dichotomous response here, with quantitative-response analysis available in the supplement.

**Fig. 2:**
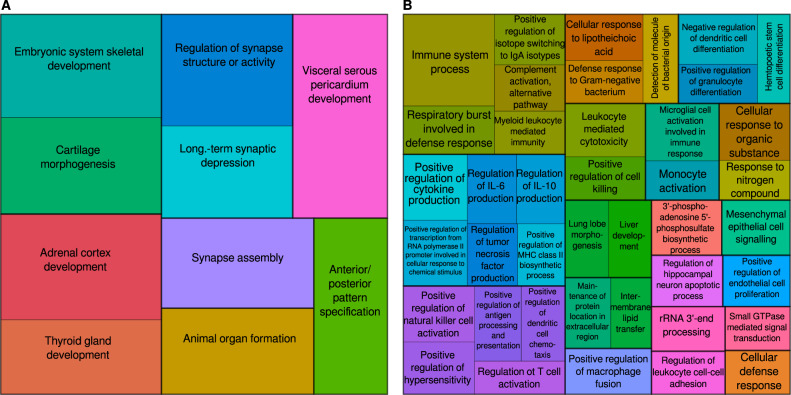
Enriched biological processes (BP) in differentially methylated regions. Regions with average methylation differences between the good and bad responders (**A**) and in those with response-specific treatment effect **(B)**. Terms were filtered to reduce redundancy and grouped by semantic similarity, with similar terms represented by shades of the same colour, and the rectangle size inversely proportional to the term’s p-value.

**Fig. 3:**
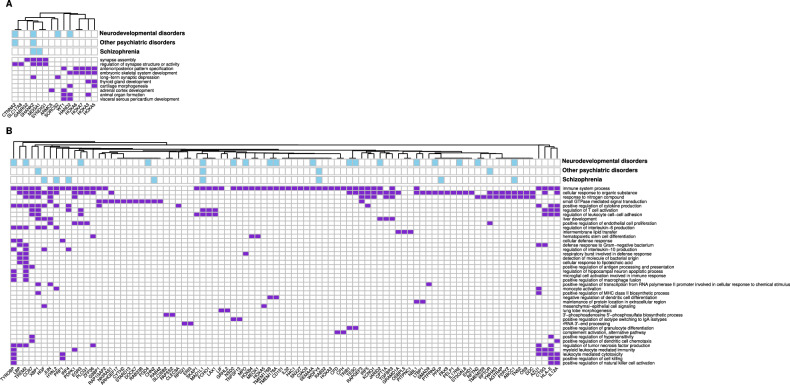
Differentially methylated genes associated with the enriched biological processes (BP). Regions with average methylation differences between good and bad responders are shown in **A** and those with response-specific treatment effect in **B**. Genes annotation at the top of the heatmaps show if the gene has previously been associated with schizophrenia, other psychiatric disorders or neurodevelopmental and neurodegenerative conditions based on the ClinVar, OMIM, MedGen and GWAS catalog databases.

The processes enriched in the regions differentially methylated between the good and bad responders included morphogenesis, synapse assembly and neurotransmission, whereas the regions with a response-specific treatment effect were mostly involved in inflammation, cell signaling and development (Fig. 2, Supplementary Table S6). Some identified genes have known links with psychiatric, neurodevelopmental and neurodegenerative conditions, including schizophrenia (Fig. 3, Supplementary Table S7, Supplementary Fig. S3). This included the genes explicitly involved in neuronal development and neurotransmission (e.g. *MDGA1* and *SHANK2* in synapse assembly), but also those annotated to less specific terms (e.g. *CHN2* in small GTPase signal transduction or *EXOC2* in cytokinesis).

### Methylation in response-associated regions correlates with the expression of schizophrenia and antipsychotic drug response-associated genes

Expression level of 1141 genes correlated with methylation in at least one response-related region on at least one sampling date (Supplementary Table S8). Of these, 149 genes were influenced by a single region or a CpG site, the rest with at least two sites (median [IQR] = 5 [4, 7]). Both CpG and region-level analysis revealed the enrichment of purine biosynthesis-related processes for the genes whose expression correlated with methylation in the regions significant for the main effect of response. Conversely, the terms enriched in the response-specific treatment effect group, varied depending on the analysis level (Supplementary Fig. S4, Supplementary Table S9), suggesting that a multilevel analysis could uncover complementary mechanisms of antipsychotic response. Finally, 519 of these gene overlapped with the genes dysregulated in either autism, schizophrenia, bipolar disorder, depression or alcoholism [34], with a marginally significant overrepresentation for the schizophrenia group (Fisher’s exact test OR [95%CI] = 1.3 [1.04, Inf], *p* = 0.02, Supplementary Fig. S5).

### Baseline methylation patterns of HOXA, HTR2A and PRR5 genes predict amisulpride response

**Fig. 4:**
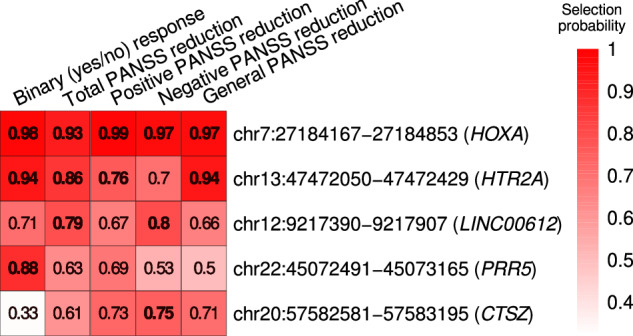
Heatmap showing the selection probabilities for the regions with baseline methylation values predicting different components of treatment response. Probabilities >= 0.75 were considered significant and are shown in bold. Region coordinates are based on GRCh37.

Variable selection procedure including baseline methylation patterns of the 467 candidate regions and response-associated patient traits (Supplementary Table S1) retained only methylation values of three regions, located in *HOXA, HTR2A* and *PRR5* genes, as predictors of amisulpride response (model performance: 10× cross-validated Kappa = 0.81, MCC = 0.81, AUC = 0.89). Note that the results for total and PANSS subscores reduction models were similar (Supplementary Table S10, Fig. 4), but characterized by poorer model performance (R2 = 0.29–0.47, Supplementary Table S11). Excluding the individuals of non-European ancestry yielded similar results, with an additional *HOXA-*associated region selected as an antipsychotic response predictor and a slightly higher performance (MCC = 0.85, Kappa = 0.84, AUC = 0.90, Supplementary Table S11). Further details, including the confusion matrix for binary response and correlation between the observed and predicted values for PANSS reduction can be found in Supplementary Figs. S6–S8 and Supplementary Table S10.

### Influence of genetic factors on the epigenetic biomarkers varies between the regions

**Fig. 5:**
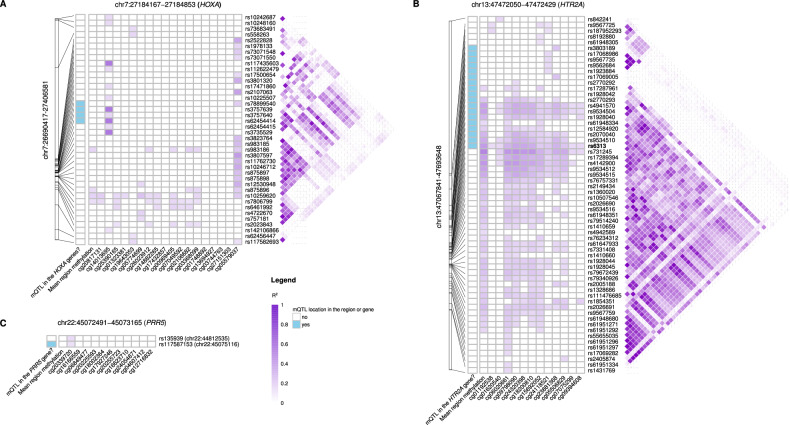
Association (R^2^) between the methylation in the response predicting regions and their corresponding mQTLs. **A** *HOXA*, **B** *HTR2A* and **C** *PRR5*. The association was calculated for each CpG in a region separately and for the mean region methylation value. LD = 1 shows the number of SNPs in complete linkage for each mQTL, LD > 1 shows the number of linked SNPs (LD > 0.8) excluding those in complete linkage. “mQTL in the region” shows if a particular SNP is located in the response-related co-methylated region, “mQTL in the same gene as the region?” marks the SNPs that are annotated to the same gene as the corresponding co-methylated region. Counts in annotation columns are capped at 7 and 35 (based on 75% quantile) for clarity. Genomic coordinates are based on GRCh37. SNP rs6313 in **B** is shown in bold.

We identified mQTLs within 1MBp window around each of the three response-predicting regions, ranging from two mQTLs affecting a single CpG in the *PRR5* region, to 62 mQTLs and a majority of CpGs influenced by multiple polymorphisms in the *HTR2A* region (Fig. 5, Supplementary Table S12). Two *HTR2A* SNPs, rs6313 and rs6311 (here in complete LD), have been previously linked with variation in antipsychotic response [35]. Although we found no deviation from the expected number of mQTLs given the number of tested SNPs in either *HTR2A* or *HOXA* region (Fisher’s exact p-values = 0.233), the number of CpGs affected by the mQTLs and the number of mQTLs affecting each CpG varied considerably between the two regions. In *HTR2A*, CpGs were affected by a median of 23 mQTLs (IQR 7.5–36.5), compared to 3.5 mQTLs (IQR 0.3–4.8) for the *HOXA* region. Correspondingly, a single mQTL affected a median of 4.5 CpGs in *HTR2A* region (IQR 1–12), and a single mQTL (IQR 1-1) per CpG in *HOXA* region. Characterization of these mQTLs or linked SNPs (R2 > = 0.8) using the LDlink database revealed two over-represented traits: “pulse pressure” and “waist circumference adjusted for body mass index” (BH-corrected Fisher’s exact test p-value < 0.05), with other high-ranked traits also related to weight and circulatory system (Supplementary Fig. S9, Supplementary Table S13).

### Genetic and epigenetic variation of response-predicting regions correlates with expression of genes involved in nervous system functioning

For each response-predicting region, we identified at least two genes whose expression covaried with either whole (mean) region methylation or with the methylation at its constitutive CpG sites (Supplementary Table S9, Supplementary Fig. S10). However, these correlations were mostly weak and unstable over time and across individual CpGs. For example, a weak negative correlation (rmcorr [95%CI] = −0.07 [−0.12, −0.03], not shown) between the expression and methylation of *HTR2A* gene at baseline turned into a positive correlation at follow-up (rmcorr [95%CI] = 0.14 [0.08, 0.22]). In addition, mean methylation of this region, and the CpG cg23881368 in particular, were negatively correlated with the expression of *LRCH1*, a regulator of microglia-mediated neuroinflammation, at baseline only (rmcorr [95%CI] = −0.13 [−0.17, −0.06] and Spearman’s rho [95%CI] = −0.22 [−0.39, −0.07], respectively). Methylation of *HOXA* region correlated with the expression of several *HOXA* genes, varying in magnitude and direction, contrary to rather stable negative correlations with *SKAP2* expression, a gene involved in src signaling – implicated, among other, in neuroinflammation [36]. Finally, the correlations in the *PRR5* region were more consistent, especially at the mean region methylation level, reveiling a stable positive correlation with the expression of *LDOC1L* and a negative one with the expression of *NUP50*, a gene predicting the risk of Alzheimer disease [37].

## Discussion

The ability to predict response to antipsychotic treatment in individual cases would help reduce patients’ suffering and alleviate pressure on the healthcare systems. A way to increase our odds in the search for reliable biomarkers is to improve our understanding of how levels of biological variation interact to shape the treatment response. Our analysis of blood methylomes of amisulpride-treated first-episode psychosis patients revealed multiple associations between methylation on one side and antipsychotic response and gene expression on the other. Moreover, we were able to predict the response from the baseline methylation of three regions with good reliability in our cohort. Finally, by integrating ours and publicly available data from genetic to gene expression level, we could show that these candidate epigenetic biomarkers were under varying degree of genetic control and that many of the response-associated genes were involved in psychiatric disorders or neurodevelopment. Our results suggest that variation in treatment response arises through an interplay of genetic, developmental and environmental factors. Whereas this conclusion corroborates the results of previous studies based on genetic [38] and epigenetic data [13] or their combination [39], the identified genes and regions mostly differ, likely due to differences between the cohorts, tested antipsychotics and experimental designs [12–15].

Schizophrenia manifests itself by a wide spectrum of symptoms, occurring with variable intensities and in different combinations and antipsychotics may differ in efficacy against particular symptom subgroups [40]. Although the regions predicting improvement of specific symptom groups mostly overlapped, we did observe some differences. For example, only negative PANSS reduction was predicted by methylation in *LINC00612*, a non-coding RNA involved in inflammation and apoptosis [41], but not by *HTR2A* methylation. However, non-significant selection probabilities were mostly close to the threshold in all models and the candidate regions were selected based on their link with total response in univariate analysis, and therefore we might have missed regions specific for individual subscores. Still, positive correlations between the PANSS subscores observed here (not shown) and elsewhere [14] suggest pseudospecificity [42] and therefore biological explanation for the observed overlap.

The major response predictor, for both overall response and symptom subgroups, was a region annotated to several *HOXA* genes, vital for the development of nervous system [43], but also involved in neurodegeneration [44]. Interestingly, *HOXA3* gene was associated with response to risperidone in our previous study [13]. *HOXA* methylation may reflect both genetic factors and early life environment. Here, we observed little evidence of *cis* genetic control of *HOXA* methylation, which, together with the evidence of its environmentally-driven methylation in the adulthood [45] and a life-long influence of *HOXA* genes on synaptogenesis [46], suggests that this region may also tag lifestyle or other environmental determinants of treatment response. This is in contrast with *HTR2A* region, where methylation patterns were largely attributable to genetic polymorphisms.

The association between treatment response and *HTR2A* methylation here may seem surprising, given the very low affinity of amisulpride for serotonin receptors [47]. Nevertheless, monoaminergic neurotransmitter systems interact with each other [48, 49] and *HTR2A* can modulate effects of dopamine antagonists, such as haloperidol [50, 51]. Similarly, interaction of *DRD2* (rs1076560) and *HTR2A* (rs6314) polymorphisms affects response to olanzapine [52]. Of note, although our sample size was insufficient to test this interaction, none of the good responders was a homozygote for a minor rs6314 allele (Fisher’s exact text *p* = 0.03). In addition, although we could not link rs6314 to methylation here, two other *HTR2A* SNPs, rs6311 and rs6313, associated with response to haloperidol [53], amisulpride [54], risperidone and olanzapine [35], acted as mQTLs in our cohort, suggesting that methylation may mediate their effects. However, further steps along the cascade leading to phenotypic variation remain unclear, with the unstable correlation between *HTR2A* methylation and expression in our study mirroring previously reported inconsistent relationship between the *HTR2A* mRNA and protein levels in the blood [53]. Furthermore, whereas methylation in the *HTR2A* region predicted reduction of total, general and positive, but not negative symptoms in our cohort, these *HTR2A* mQTLs (rs6311 and rs6313) have been usually linked to the improvement of positive and negative symptoms [35, 54]. These incongruences may reflect epistatic [55] or *trans* epigenetic mechanisms, but also the complexity and interactions within neurotransmission systems [49].

*HTR2A* affects not only various aspects of mood and behaviour, but also cardiac function and digestive system [56]. Luo et al. [57] observed lower weight gain for rs6313 CC genotype carriers following antipsychotic treatment, unlike us (but we did observe high weight gain for rs6314 TT homozygotes, not shown). This pleiotropy may help explain high prevalence of antipsychotic-related side effects or schizophrenia-related comorbidities. Interestingly, the third identified region, in the *PRR5* gene is involved in mTORC2 signalling [58], a pathway mediating dopamine-dependent behaviours [59], but also the side-effects of antipsychotic drugs [60].

At least some forms of schizophrenia can be linked to inflammatory processes [61] and antipsychotics can affect innate immunity [62, 63]. Although the only difference in plasma protein levels observed here was a lower concentration of the cytokine CCL22 in bad responders, the treatment-affected methylation predominantly in the regions involved in immunity and inflammation, supporting a link between antipsychotic response and immunity. Differential expression of the genes attributable to methylation shifts in our study and the indirect evidence linking our mQTLs to gene expression from the public databases, strengthen this conclusion. These genes include: alphamacroglobulin *A2M*, a peripheral marker of neuroinflammation affecting susceptibility to major depressive disorder [64]; *A2ML1*, discriminating schizophrenic from bipolar patients and healthy controls [65], and *PZP*, a chaperone linked with Alzheimer’s disease [66].

Regarding the limitations of our study, we are aware that amisulpride is unavailable in the USA, limiting clinical utility of our findings there. However, it is approved and used to treat schizophrenia in Europe and 50 other countries. In addition, amisulpride ranks among top antipsychotics in terms of both symptom improvement (including the negative ones) and side-effects profiles according to multiple studies [17, 19, 40, 67–69]. This, with its good cost-effectiveness [70], makes it a good candidate for a broader use in the future, especially in low- and middle-income countries. Furthermore, although blood has a high clinical potential as a source of biomarkers due to easy accessibility, it remains unclear if and how methylation and expression in the blood reflect processes in the central nervous system. However, although methylation in blood and the brain co-varies only at few sites [71], blood methylome can still tag psychiatric disorders, as these co-occur with alterations in lymphocyte functions and abundances [72–74], and these are characterized by specific methylation profiles [75]. Comparing the methylation-based leukocyte composition estimates with those obtained from direct measurements could provide more insight into relative contributions of cell composition shifts and (de)methylation to methylation patterns observed in the blood. Furthermore, we did not examine *trans* regulation of epigenetic variation and gene expression. As *trans* correlations could account for non-negligible portion of genetically controlled methylation differences [76], we may have missed additional response-related processes. Regarding our cohort, 90% of the individuals are of European ancestry and the prediction models may not be straightforwardly transferable to non-European populations. For example, Ota et al. [15] have found very little overlap of the risperidone-response associated differentially methylated sites between the Brazilian and Indian cohorts. In addition, a matching validation cohort would provide a stronger support for the results than the model cross-validation we performed here. Finally, a larger cohort would have improved the robustness of the findings and increased statistical power [77] and should be preferred especially when studying multiple antipsychotics and heterogeneous populations. Still, our work represents one of the rare prospective studies aiming to identify epigenomic biomarkers of antipsychotic response by genome-wide analysis. Our approach combining a focus on a single antipsychotic, a prospective study design and a narrowly delineated cohort including only first-episode psychosis patients, with the analysis protocol accounting for all major sources of epigenetic variation (sex, tobacco use, ancestry, age) and biochemically confirmed adherence, allowed us not only to identify epigenetic biomarkers of the response to amisulpride in our cohort and shed some light on the involved pathways, but also to provide a well-defined reference for future comparisons.

## Supplementary information

Supplementary methods and figures

Supplementary Table S1

Supplementary Table S2

Supplementary Table S3

Supplementary Table S4

Supplementary Table S5

Supplementary Table S6

Supplementary Table S7

Supplementary Table S8

Supplementary Table S9

Supplementary Table S10

Supplementary Table S11

Supplementary Table S12

Supplementary Table S13

## Data Availability

Raw methylation intensity data are deposited in the ArrayExpress repository (accession number: E-MTAB-13006). Methods are described in detail in Supplementary Materials and Methods. All other data and scripts are available on Figshare or upon request.
