# Supplementary material for: Using blood methylomes to predict response to amisulpride in the first-episode psychosis in the OPTiMiSE cohort
Source: Transl Psychiatry. 2025 Oct 6;15:369. doi: 10.1038/s41398-025-03561-7 (PMC12501013; doi:10.1038/s41398-025-03561-7)
Supplement: Supplementary file 1 — Supplementary methods and figures [file 41398_2025_3561_MOESM1_ESM.pdf]

# Supplementary Material for the article “Using blood methylomes to predict response to amisulpride in the first-episode psychosis in the OPTiMiSE cohort” by Lokmer et al.

This document contains:
1) Composition of the OPTiMiSE study group
2) Supplementary figures legends
3) Supplementary tables legends
3) Supplementary methods with references
4) Supplementary figures

## Composition of the OPTiMiSE study group:

René S Kahn, Iris E Sommer, Inge Winter-van Rossum, Metten Somers, Paula C Ywema, Shitisj Kapur, Philip McGuire, Marion Leboyer, Andreas Meyer-Lindenberg, Shon W Lewis, Stefan Leucht, Celso Arango, Wolfgang W Fleischhacker, Anne Lotte Meijering, Jocelyn Petter, Resy Van de Brug, Joost Schotsman, Jildou Zwerver, Jos Peuskens, Marc De Hert, Erik Thys, Lucho G Hranov, Valentin Hranov, Jan Libiger, Richard Köhler, Pavel Mohr, Birte Glenthoj, Brian Broberg, Signe Düring, Lone Baandrup, Stéphane Jamain, Stephan Heres, Dan Rujescu, Ina Giegling, Mark Weiser, Mor Bar Heim, Michael Davidson, Silvana Galderisi, Paola Bucci, Armida Mucci, Janusz Rybakowski, Agnieszka Remlinger-Molenda, Ilan Gonen, Paull Radu, Marina Díaz-Marsá, Alberto Rodriguez, Tomas Palomo, Roberto Rodriguez-Jimenez, Paz García-Portilla, Miquel Bernardo, Julio Bobes, Christina Vilares Oliveira, Gregor Berger, Claudia Wildt, Paola Dazzan, Roccio Perez-Iglesias, Richard Drake, Sarah Gregory, Danielle Wilson, Covadonga M Díaz-Caneja, Lieuwe de Haan, Arjen Sutterland.

## Supplementary Figures Legends

**Figure S1**. Variability of leukocyte composition estimated based on the methylation data. The heatmap shows relative abundances of six leukocyte types in the individual samples (narrow columns) and their mean values for the three “leukocyte community” types (“Dirichlet components”, wide columns) identified by Dirichlet multinomial mixture modelling. All three types are characterized by high proportion of granulocytes (neutrophils) and they differ mainly by the relative abundances of CD4+ and CD8+ T-cells. Sample annotation (response and visit) is shown on the top of the figure. There is no significant correlation between the lekocyte community type and the response to amisulpride, despite the significant difference in the relative abundance of natural killer cells in the univariate tests (Table S1). The samples are ordered according to the default parameters of the plotting function (heatmapdmn in the R DirichletMultinomial package).

**Figure S2.** Overlap between the groups of regions whose methylation is significantly associated with response to amisulpride according to different criteria. The first row shows overlap between

the regions with average methylation differences between the good and bad responders (“main effect of response) and those with response-specific treatment effect (“response x visit interaction”) for the response defined as (A) dichotomous (yes/no) or (B) continuous (%PANSS improvement) variable. Panel (C) shows overlap of the regions significant for the main effect of response between the models with different response definitions (binary vs. continuous), panel (D) shows the corresponding overlap for the regions with response-specific treatment effect. Whereas the panels A-D show overlaps between the pairs of the region subsets, (E) is an upset plot showing also three-way intersections (no region was found in all four groups). Colors of the bars showing the total number of significant regions in the (E) correspond to the colors in the panels A-D.

**Figure S3.** Biological processes (BP) enriched in the regions whose methylation co-varies with the %PANSS improvement (A) and in those with response-specific treatment effect (C), with the heatmaps showing the differentially methylated genes within the respective enriched terms (B, D). Terms in A & C were filtered to reduce redundancy and grouped by semantic similarity, with similar terms represented by the shades of the same color, and the rectangle size is inversely proportional to the corresponding term p-value. Genes annotation at the top of the heatmaps (B & D) show if the gene has previously been associated with schizophrenia, other psychiatric disorders and eurodevelopmental and neurodegenerative conditions based on the ClinVar, OMIM, MedGen and GWAS catalog databases.

**Figure S4.** Biological processes (BP) enriched in the genes whose expression is correlated with the methylation of the regions whose methylation differs on average between the good and bad responders (A-B) or those with response-specific treatment effect (C-D). A & C show the results for the genes whose expression is correlated with the methylation of individual CpGs within the regions of interest, B & D show the enrichment results for the genes with expression correlating with the whole-region methylation. The results are based on topGO weight01 algorithm. Terms were filtered to reduce redundancy and grouped by semantic similarity. Rectangle size is inversely proportional to a term’s p-value.

**Figure S5.** Overlap between the genes identified by expression quantitative trait methylation analysis (eQTM) and whose expression is thus correlated with the methylation of response-associated regions, and the genes dysregulated in several psychiatric disorders according to Gandal et al., Science 2018. The upset plot shows all the intersections with eQTM-identified genes, Venn diagrams in the inset show pairwise overlap of the eQTM-identified genes with each of the psychiatric disorders separately.

**Figure S6.** Performance of predictive models. The first two rows show plots of observed *vs* predicted values for total, negative (first row), positive and general (second row) PANSS reduction, with models based on all subjects on the left and Europeans only on the right. Receiver operator characteristic (ROC) curves for all (black) and European only (red) subjects are shown in third row, with the corresponding confusion matrices in the inset.

**Figure S7**. Association (R2) between the methylation in the regions predicting %PANSS improvement and their corresponding mQTLs: (A) LINC00612 and (B) CTSZ. The association was calculated for each CpG in a region separately and for the mean region methylation value. LD = 1

shows the number of SNPs in complete linkage for each mQTL, LD > 1 shows the number of linked SNPs (LD > 0.8) excluding those in complete linkage. "mQTL in the region" shows if a particular SNP is located in the response-related co-methylated region, "mQTL in the same gene as the region?" marks the SNPs that are annotated to the same gene as the corresponding co-methylated region. Counts in annotation columns are capped at 7 and 35 (based on 75% quantile) for clarity. Genomic coordinates are based on GRCh37. SNP rs6313 in (B) is shown in bold.

**Figure S8**. Correlations detected between the gene expression and methylation of the PANSS reduction predicting region LINC00612 in this study (A) and the effect of the mQTLs associated with this region on the gene expression in the blood and brain tissues according to GTEX8 database (B). Correlations were calculated for the mean methylation value of the region (Spearman’s rho), for the whole region accounting for the variability of individual CpGs within the region (rrcor) and for the individual CpGs in the region (Spearman’s rho), for both visits together and for each visit separately. Rectangle size is proportional to the strength of correlation, the thickness is proportional to the width of confidence intervals. For (B), association with the gene expression is shown only if the SNP affects the expression of the gene in both blood and brain. The effect sizes are slopes of the linear regression, computed as the effect of the alternative allele relative to the reference one according to the GTEX portal FAQ. The size of the circle is proportional to the effect size.

**Figure S9.** Wordcloud of over-represented (raw Fisher’s exact p-value <= 0.05) GWAS traits associated with the SNPs that act as mQTLs for the HOXA and HTR2A co-methylated regions. Over-representation of the GWAS traits associated with the mQTLs was calculated relative to the traits associated with the set containing all SNPs tested for the link with methylation. SNPs that are in the linkage (R 2 > 0.8) with the tested SNPs were also included. The colors are chosen by the software randomly and have no meaning except to increase readability. The font size is proportional to the frequency of the trait in the gene set.

**Figure S10.** Correlations between the gene expression and methylation of the binary response (yes/no) predicting regions detected in this study: A) HOXA, C) HTR2A and E) PRR5, and the effect of the mQTLs associated with these regions on the gene expression in the blood and brain tissues according to GTEX8 database: B) HOXA, D) HTR2A. Correlations were calculated for the mean methylation value of the regions (Spearman’s rho), for the whole regions accounting for the variability of individual CpGs within the regions (rrcor) and for the individual CpGs in the regions (Spearman’s rho), for both visits together and for each visit separately. Rectangle size in A, C & E is proportional to the strength of correlation, the thickness is proportional to the width of confidence intervals. For B & D, association with the gene expression is shown only if the SNP affects the expression of the gene in both blood and brain. The effect sizes are slopes of the linear regression, computed as the effect of the alternative allele relative to the reference one according to the GTEX portal FAQ. The size of the circle is proportional to the effect size.

## Supplementary Tables Legends

**Table S1**. Tested pairwise differences in the clinical and anthropometric traits between the good and bad responders. For the continuous traits, we first perfromed the Levene's test of homogeneity of variances. If the variances were homogenous across the groups, we performed a Welch Two Sample t-test and a Wilcoxon-Mann-Whitney test if they were not. For the categorical variables, we employed Fisher's exact test for count data. Post-hoc pairwise tests were performed if the main effect test was significant (e.g. for sampling centre) and these are shown, if significant, in italics. Significant differences are ordered according to the obtained raw p-values, the rest is grouped by trait type (e.g. immunological). Non-significant values are greyed out. VA = baseline, VB = follow-up.

**Table S2**. Regions with methylation values significantly associated with the main effect of response (average difference between good and bad responders) for the response defined as dichotomous variable (good vs. bad responders). The models included covariates (sex, age, tobacco use, drug abuse, leukocyte composition and technical variation). Three regions that are significant for visit x response interaction as well are shown in bold.

**Table S3**. Regions with methylation values with significant response-specific treatment effects (visit x response interaction) with the response defined as dichotomous variable (good vs. bad responders). The models included covariates (sex, age, tobacco use, drug abuse, leukocyte composition and technical variation). Three regions that are significant for the main effect of response as well are shown in bold.

**Table S4**. Regions with methylation values significantly associated with the main effect of response (covarying with %PANSS improvement) for the response defined as % total PANSS improvement. The models included covariates (sex, age, tobacco use, drug abuse, leukocyte composition and technical variation). Ten regions significant for both the main effect of response and response x visit interaction are shown in bold.

**Table S5**. Regions with methylation values with significant response-specific treatment effects (visit x response interaction) with the response defined as % total PANSS improvement. The models included covariates (sex, age, tobacco use, drug abuse, leukocyte composition and technical variation). Ten regions significant for both the main effect of response and response x visit interaction are shown in bold.

**Table S6**. Biological processes enriched in regions significant for response or response x visit interaction. Response is defined either as a dichotomous (good/bad) or continuous variable (%PANSS improvement). Background comprises all tested regions. Raw enrichment results were analyzed using GOxploreR to identify the most informative terms (column "Prioritized") and the prioritized terms were grouped according to semantic similarity ("Cluster" columns, for details see Methods).

**Table S7**. Genes in the enriched GO BP terms for which we found evidence of association with schizophrenia, other psychiatric diseases or neurodevelopemental and neurodegenerative disorders in ClinVar, MedGen, Omim or GWAS database or in the NCBI gene summaries. We filtered the hits

using case insensitive search for “scz” (schizophrenia), “depres|bipol|obsess| mani|affectiv” (other psychiatric diseases) or “autis|Alzhei|dement|neurodeg|neurodev|Parkins” (neurodevelopmental and neurodegenerative disorders) and manually inspected the results.

**Table S8**. Significant cis (<500kb distance) correlations between gene expression and methylation of the regions associated with response variation (either of the region as a whole or of the individual CpGs within). For the whole regions, correlations were calculated either as rrcor, accounting for the variability of the individual CpGs in the region or as Spearman’s rho, for a simple mean region methylation value. Correlations between individual CpGs and gene expression are represented by Spearman’s rho. Only the correlations with a confidence interval at least 0.05 far from 0 are shown (i.e. the lower CI of positive correlations >= 0.05 and the upper CI of negative correlations <=-0.05). Brain-blood correlation for methylation values from the Image-CpG tool are shown where available.

**Table S9**. Biological processes enriched in the genes whose expression was correlated with methylation of response-related regions at either whole region or CpG level. For details see Methods and Table S9.

**Table S10**. Stability selection for different response definitions. The numbers represent selection probabilities. Selection probability cutoff (q) was set at 0.75. Predictors selected for at least one response definition are shown in bold. The columns on the left show the results with the individuals of non-European ancestry excluded.

**Table S11**. Model performance for the response-predicting models based on the variables selected by stability selection procedure. The values are R2 for continuous responses, and Matthews correlation coefficient (MCC) for binary response. Chromosome position are based on genome assembly 37 (GRCh37).

**Table S12**. Association between genetic variants and methylation in the regions predicting amisulpride response (binary or PANSS improvement), including the associations between the mQTLs (SNPs significantly covarying with methylation) and the gene expression in the brain and blood according to the GTEX v8 database. SNPs in a 1MB window around the region were tested for association with methylation of either individual CpGs in a region or the region mean methylation value. For the SNPs in complete linkage (LD R2 = 1) with each other, only one SNP per group was tested. If the mQTLs or the SNPs linked with them (LD R2 >= 0.8, LDlink) consistently affect the gene expression (eQTLs) in the blood and brain tissues according to the GTEX v8 database, the names of the associated expressed genes and the corresponding effect sizes for the brain and blood are listed. For the brain, the effect sizes correspond to the median effect size with MAD (median absolute deviation) if the expression is affected by eQTLs in multiple brain tissues. Tested SNPs with no significant associations are listed at the end of the table for completeness.

**Table S13**. GWAS traits associated with the mQTLs affecting the methylation of the treatment-response predicting regions or the SNPs linked with them (R2 > 0.8) in the European superpopulation retrieved from linkLD database.

## Supplementary methods

*Participants and study design*

The cohort analyzed here represents a subset of patients enrolled in the OPTiMiSE (Optimization of Treatment and Management of Schizophrenia in Europe, NCT01248195) clinical trial (1). It comprises 116 adults (41 women and 75 men) recruited in 18 hospitals across twelve European countries and experiencing the first episode psychosis (FEP) for no longer than two years. Only individuals whose gender identity corresponded with their genetic sex were included in the study. The participants had either never been treated with antipsychotics (AP) or had been treated for a maximum of two weeks in the previous year or for a total of six weeks in their lifetime before the beginning of the experiment, when they were prescribed 200-800 mg/day amisulpride orally Amisulpride was chosen based on its comparatively good efficacy and relatively narrow side-effects profile (2,3). Compliance with treatment was confirmed by measuring the amisulpride concentration in the blood.

The participants were diagnosed with schizophrenia or related disorder (schizophreniform or schizoaffective disorder) according to the Diagnostic and Statistical Manual of Mental Disorders, Fourth Edition (DSM-IV). Symptom severity was assessed using the total Positive and Negative Syndrome Scale (PANSS) score at the baseline, just before the beginning of the treatment, and four weeks later during the follow-up visit. As described previously (4), patients with at least 20% reduction of PANSS score at the follow-up were classified as (good) responders, while those with PANSS reduction below this threshold were considered as bad or non-responders (Figure 1).
A written informed consent was obtained from all participants prior their inclusion in the study. All inclusion sites obtained ethical approval and this research was approved by an Institutional Review Board.
Blood for the methylome analysis, genotyping, gene expression and immune parameter measurements was collected during both visits. Genotyping revealed that 104 patients were of European, eight of Asian and four of African origin and the first three genetic principal components (PCs) were used in statistical analyses to account for genetic diversity at population level (i.e. ethnicity). No further data regarding the participants' ethnicity were collected. Cytokine levels were measured due to their potential involvement in schizophrenia treatment response (for review see (5). Additional details regarding genotyping, gene expression and cytokine measurements had already been published and can be found in (1,4,6),

*Blood processing and generation of raw methylome data*

Genomic DNA was extracted from whole blood in EDTA tubes or buffy coat samples using the Maxwell® 16 Blood DNA Purification Kit (Promega Corp., Madison, USA) according to the manufacturer's standard protocols. The amount of DNA was measured in fluorescence, in duplicate, using Quant-iT™ dsDNA Assay Kit, broad range (ThermoFischer Scientific) and a SpectraMax M3 fluorescence microplate reader from Molecular Devices. DNA quality control was performed by

checking the absence of PCR inhibitors and evaluating the DNA integrity by electrophoresis migration (4200 TapeStation, Agilent Technologies Inc., Santa Clara, CA, U.S.A.). All samples had a DNA integrity number (DIN) higher than 7. Bisulfite conversion of 1µg of genomic DNA was performed using EpiTect Fast 96 Bisulfite Kit (Qiagen, Germany) and genome-wide methylation was assessed using Infinium Methylation EPIC v1.0 BeadChip on the Centre National de Recherche en Génomique Humaine (CNRGH) automated platform, following the manufacturer’s protocol (Illumina Inc., USA).

*Quality control and normalization of methylome data*

All analyses and data visualization were performed in R statistical framework version 4.1.3 (7). The quality control and normalization were conducted following recommendations of (8), as described in full detail in (9). Briefly, we removed low-quality probes and samples, and peformed background (*"noob"*, (10), dye-bias ("RELIC", (11) and design-bias ("BMIQ", (12)) normalization steps. At least one paired sample in eleven individuals did not pass the quality control and the cleaned and normalized dataset comprised 210 paired samples (i.e. 105 individuals with both data points) and 785,997 good quality probes.

*Estimation of leukocyte composition and batch effects*

As each leukocyte type has specific methylation profile, adjusting for the leukocyte type composition is necessary to prevent confounding with other methylation-influencing factors (13). We estimated relative abundances of six cell types (neutrophils/granulocytes, CD4+ and CD8+ T-cells, B-cells, monocytes and NK cells) using the *flow.sorted.blood.EPIC* v. 1.10.1 package (14). We further applied Dirichlet-multinomial mixture (DMM) modelling (15) implemented in the *DirichletMultinomial* v. 1.40.0 package (16) to the estimated leukocyte compositions. In this way, we reduced the number of covariates accounting for the cell type composition from six to a single categorical variable with three levels, thereby reducing the probability of overfitting and artifacts during statistical analysis. The three DMM-detected profiles differed mainly by the relative abundances of CD4+ and CD8+ T-cells (Figure S1).
Similarly, to account for technical variation while avoiding to include too many covariates in the statistical models, we analyzed technical variation by factor analysis (FA) using the FactoMineR package v. 2.8 (17). The analyzed variables included sampling site, chip, position on the chip, sample processing plate, row and column on the processing plate and the "control matrix" calculated from the methylation data (18). We selected the first three FA axes (accounting for 17 + 7.7 + 7 = 31.7% of variation) to represent the batch effects in the subsequent analyses. The procedure is described in detail in ((9).

*Detection of co-methylated regions*

Methylation status at individual sites is often spatially correlated and we therefore opted for the region-based analysis in our study, due both to higher statistical power and increased reproducibility compared with the single-site based approaches (19). We identified co-methylated regions in our dataset using the unsupervised method of (20) implemented in the *coMethDMR* v.1.2.0 package. A co-methylated region was defined as a cluster of at least three CpGs, with a maximum distance of 200 bp between the consecutive probes and a minimum correlation of 0.4 between the beta value of a CpG and the mean methylation of all other probes in a given region. We identified 16,118 autosomal regions including a total of 63,291 CpGs (mean number of CpGs per region = 3.93, median = 3.00, IQR = [3.00, 4.00], maximum = 32).

*Detection and functional characterization of differentially methylated regions*

In order to identify regions associated with variation of the amisulpride treatment response, we adapted the random coefficient mixed effects model method from the *coMethDMR* package (20) to our study design. Specifically, we fitted the models using the *glmmTMB* v.1.1.2.3. package (21) with beta family and *logit* link instead of *lmer* Gaussian family method (22), as beta values are bounded between 0 and 1 and, in addition, beta distribution allows various distribution shapes, reflecting more closely those of methylation values than the commonly used normal distribution (23,24). We assessed associations between the methylation value of each region (dependent variable) and amisulpride response defined either as dichotomous outcome (categorical predictor) or as %PANSS reduction (numerical predictor). We were interested in both stable methylome differences between the good and bad responders (main effect of response) as well as in response-specific temporal shifts following AP treatment (response x visit interaction). Taken the heterogeneity of our cohort and the well-recognized influence of the factors such as genetics (25), smoking (26), age (27), sex (28) and blood cell type composition (13) on methylation, the models included following covariates: age, sex, smoking, alcohol use, body mass index (BMI), duration of untreated psychosis (DUP), leukocyte composition, first three genetic PCs and the first three batch effects PCs. Drug use was not included, as it was strongly correlated with tobacco use ($\chi^2 = 41.59$, df = 2, $p < 10^{-10}$, Cramer's V = 0.599). Missing values were imputed using the *missForest* v. 1.5 package (29). Including of patient, sample and probe IDs (intercept only for the first two and both the intercept and amisulpride-response related slope for the probe IDs) as random effects allowed us not only to account for repeated measures design, but also for the methylation variability and signal consistency across the region (20). Before evaluating relationship between methylation and AP response, we removed the models with multicollinearity, convergence and/or issues related to outliers, uniformity and dispersion (30). Good quality models were compared to the corresponding null models with a likelihood ratio test (LRT) and the obtained p-values were corrected for multiple testing using the Benjamini-Hochberg procedure. We selected all significant coefficient estimates (p-value < 0.05) from the models with the adjusted LRT p-value < 0.05 for the subsequent analysis. To summarize functional aspects of the identified regions, we performed a region-set enrichment analysis using *goregion* method, specially adapted for the methylation array data (31). The region/CpG universe comprised all tested autosomal regions (63, 291 CpGs in 16,118 regions). As multiple testing correction is not straightforward in a multi-step procedure such as gene-set

enrichment analysis and could result in overly conservative results, we followed the advice of (32) and used term-ranking combined with raw p-values (p <0.01) to select potentially interesting terms. We also filtered out the terms with a single differentially methylated gene as well as the terms assigned to more than 2000 genes. We further applied prioritization (*GOexploreR* v 1.2.4 (33) and grouping by semantic similarity (0.7 similarity threshold, Resnik method, *GOSemSim* v.2.18.1 (34) to simplify the results and maximize their information content.
In order to examine if the observed methylation differences translated into differences in phenotype, more specifically, if methylation covaried with gene expression, we performed expression quantitative trait methylation (eQTM) analysis. To do so, we calculated repeated-measures correlations (accounting for the CpG-level variability within a region, *rmcorr* package v. 0.54, (35,36) between the region methylation values and gene expression using transcriptomes generated in (4) for all the region-gene pairs on the same chromosome, with a maximum distance of 500 kbp between the region and the gene transcription start site. Correlation confidence intervals were calculated based on 1000 resamples and we kept only the correlations with confidence intervals at least 0.1 distant from 0. In a corresponding way, we calculated Spearman's correlations between gene expression and either the individual CpGs or the region mean methylation. We calculated correlations for both visits combined and for each visit separately. We then performed GO enrichment analysis of correlated genes using weight01 algorithm (37), accounting for the topology structure and implemented in the *topGO* v. 2.54.0 package (38).
Finally, we wanted to know if any of the differentially methylated genes involved in the enriched functions identified by *goregion* have been previously associated with schizophrenia or other psychiatric, neurodevelopmental or neurodegenerative disorders. To do this, we downloaded their respective RefSeq gene summaries (39), as well as related ClinVar (40), MedGen and OMIM (41) entries (downloaded on October 29th 2023). Similarly, we compared the list of the genes whose expression was correlated with methylation in our study with the list of the genes dysregulated in schizophrenia and other psychiatric disorders identified by (42).

*Amisulpride response prediction from the pre-selected co-methylated regions*

In order to identify epigenomic biomarkers predicting response to amisulpride treatment, we used baseline methylation values of the regions selected in the previous step as input for the generalized additive modelling fitted by gradient boosting implemented in the *mboost* v. 2.9-5 package (43). To simplify the model and its transferability to other datasets, we first adjusted the values for the methylation-influencing factors (sex, age, leukocyte composition, genetic PCs 1-3) and batch effects (EPIC chip ID and position on the chip) using the *sva* procedure (*sva* package v. 3.48.0, (44,45). We also added randomly generated variables to the models as an additional check for the false positive results. We were interested in the prediction of dichotomous response and % total PANSS reduction, but also in the % reduction of PANSS subscores (positive, negative and general). We therefore repeated the procedure for each of the response definitions separately, with base learners (independent variables) including all baseline methylation values of the regions selected in the differential methylation detection step described above. As the concentration of the cytokine CCL22 concentration and sampling centre were significantly correlated with the dichotomous

response in univariate tests (Table S1), we included them in the models, in addition to the corresponding baseline PANSS sub(scores) and amisulpride dose and blood concentration (which were marginally lower in good responders, Supplementary Table S1). Binomial models with logit link function were run for dichotomous response and robust Huber regression for PANSS reduction. In order to decrease false positive rate, the models were subjected to the stability variable selection procedure (46,47), with 0.75 selection probability cutoff and upper bound for the per-family error-rate PFER = 1. We built the final predictive models by the gradient boosting modelling procedure described above combined with 10x cross-validation, using only the regions selected in the stability selection step. We used $R^2$ for assessing the performances of the PANSS reduction models, and Matthews correlation coefficient (MCC) for dichotomous response. We have chosen MCC over the more commonly used area under the curve metric (AUC), because it is more adapted for unbalanced designs (in our case there is 28% of bad responders) and for the cases where we are interested in both positive and negative predictive value of the model (48,49).

*Detection of methylation quantitative trait loci (mQTLs) associated with the co-methylated regions predicting amisulpride response*

We further wanted to know if and to what extent the regions predicting the amisulpride response were under genetic control. To identify potential methylation quantitative trait loci (mQTLs) associated with methylation in the selected regions, we first genotyped patients using the OmniExpressExome 8v1-4 A1 BeadChip (Illumina Inc.) and performed genotype quality control using PLINK v1.90b7 (50,51) as described elsewhere (Ter Hark *et al*., 2020). We then expanded genotyping data using the Michigan Imputation Server (52). Phasing and imputation were conducted using Eagle v 2.4 (53) and minimac4 (52), respectively, using the HRC r1.1 2016 reference panel. After imputation, only biallelic SNPs with a MAF higher than 0.01 in the reference panel and an imputation score higher than 0.9 were used. We imported the genotyping data into R using the s*npStats* package v. 1.50.0 (54). We removed a sample with a mismatch between the estimated and real sex and selected SNPs within a 1MB window around the response-predicting regions for association testing. We grouped perfectly collinear SNPs (linkage $R^2 = 1$) together and analyzed only one representative from each group. We fit linear models using generalized least squares (*gls* in the *nlme* v. 3.1-163 package ((55) with genotype as an explanatory variable and covariate-corrected methylation value as a dependent variable. We ran the models for both the mean methylation value of a region and for each CpG in the region separately. Weights specifying the variance for each allele were included if necessary (if the model with variance-specifying weights was better than the model without weights according to the likelihood ratio test, $p < 0.05$). The models were run for the baseline visit only as well as for both days together (in the latter case including the patient as a random effect), but the coefficient estimates were highly correlated (Pearson's $r = 0.90$, $p < 10^{-6}$) and we therefore present only the baseline visit models for simplicity. P-values were adjusted using Benjamini-Hochberg correction and we kept only those with the adjusted $p < 0.05$. We used R-squared as a measure of effect size.
To characterize SNPs surrounding the selected regions and the identified mQTLs into more detail, we calculated linkage disequilibrium (LD) R-squared for all pairs of SNPs flanking the selected

regions with PLINK v1.90b7 (50,51). In addition, we checked if the identified mQTLs acted also as expression quantitative trait loci (eQTLs), by performing a corresponding analysis with the gene expression as a dependent variable instead of methylation. Finally, we used the *LDlinkR* package v. 1.30 (56) to query the publicly available databases using the LDlink tools (57) in order to characterize the selected mQTLs or the SNPs linked with them (LD R2>=0.8) in more detail. To be specific, we downloaded a list of SNPs linked with our mQTLs from dbSNP database (58), and checked if our mQTLs were previously identified as eQTLs in healthy blood and brain tissues in GTEX v7 (downloaded on September 22$^{nd}$ 2023) or if they or the SNPs linked with them had been previously associated with psychiatric diseases or nervous system development and functioning according to the GWAS catalog v 1.0.2 (59). We downloaded these data for “EUR” superpopulation, as 90% our participants were of European origin. Where applicable, we checked the congruence of the downloaded data with the results obtained directly from our cohort. We also compared GWAS traits associated with our mQTLs with all SNPs in the queried 1MB region surrounding to find over-represented traits using Fisher’s exact test.

*Data availability*

Raw methylation intensity data can be found in the ArrayExpress repository under the accession number E-MTAB-13006. All other relevant data and scripts that are not included as a supplementary material are available on Figshare or upon request.

**References**

1. Leucht S, Winter-van Rossum I, Heres S, Arango C, Fleischhacker WW, Glenthøj B, et al. The optimization of treatment and management of schizophrenia in Europe (OPTiMiSE) trial: rationale for its methodology and a review of the effectiveness of switching antipsychotics. Schizophr Bull. 2015 May;41(3):549–58.

2. Kahn RS, Fleischhacker WW, Boter H, Davidson M, Vergouwe Y, Keet IP, et al. Effectiveness of antipsychotic drugs in first-episode schizophrenia and schizophreniform disorder: an open randomised clinical trial. The Lancet. 2008 Mar 29;371(9618):1085–97.

3. Leucht S, Cipriani A, Spineli L, Mavridis D, Örey D, Richter F, et al. Comparative efficacy and tolerability of 15 antipsychotic drugs in schizophrenia: a multiple-treatments meta-analysis. The Lancet. 2013 Sep 14;382(9896):951–62.

4. Troudet R, Ali WBH, Bacq-Daian D, Rossum IW van, Boland-Auge A, Battail C, et al. Gene expression and response prediction to amisulpride in the OPTiMiSE first episode psychoses. Neuropsychopharmacology. 2020 Sep;45(10):1637–44.

5. Fond G, d’Albis MA, Jamain S, Tamouza R, Arango C, Fleischhacker WW, et al. The Promise of Biological Markers for Treatment Response in First-Episode Psychosis: A Systematic Review. Schizophr Bull. 2015 May 1;41(3):559–73.

6. Martinuzzi E, Barbosa S, Daoudlarian D, Bel Haj Ali W, Gilet C, Fillatre L, et al. Stratification and prediction of remission in first-episode psychosis patients: the OPTiMiSE cohort study. Transl Psychiatry. 2019 Jan 17;9(1):1–13.

7. R Core Team. R: A Language and Environment for Statistical Computing [Internet]. Vienna, Austria: R Foundation for Statistical Computing; 2020. Available from: http://www.R-project.org/

8. Dedeurwaerder S, Defrance M, Bizet M, Calonne E, Bontempi G, Fuks F. A comprehensive overview of Infinium HumanMethylation450 data processing. Brief Bioinform. 2014 Nov 1;15(6):929–41.

9. Lokmer A, Alladi CG, Troudet R, Bacq-Daian D, Boland-Auge A, Latapie V, et al. Risperidone response in patients with schizophrenia drives DNA methylation changes in immune and neuronal systems. Epigenomics. 2023 Mar 15;epi-2023-0017.

10. Triche TJ, Weisenberger DJ, Berg DVD, Laird PW, Siegmund KD. Low-level processing of Illumina Infinium DNA Methylation BeadArrays. Nucleic Acids Res. 2013;41(7):e90.

11. Xu Z, Jack AT, Sabine ASL, Patrick DB, Niu L. RELIC: a novel dye-bias correction method for Illumina Methylation BeadChip. BMC Genomics. 2017;

12. Teschendorff AE, Marabita F, Lechner M, Bartlett T, Tegner J, Gomez-Cabrero D, et al. A beta-mixture quantile normalization method for correcting probe design bias in Illumina Infinium 450 k DNA methylation data. Bioinformatics. 2013 Jan 15;29(2):189–96.

13. Jaffe AE, Irizarry RA. Accounting for cellular heterogeneity is critical in epigenome-wide association studies. Genome Biol. 2014 Feb 4;15(2):R31.

14. Salas L, Koestler DC. FlowSorted.Blood.EPIC: Illumina EPIC data on immunomagnetic sorted peripheral adult blood cells. [Internet]. 2020. Available from: https://github.com/immunomethylomics/FlowSorted.Blood.EPIC

15. Holmes I, Harris K, Quince C. Dirichlet Multinomial Mixtures: Generative Models for Microbial Metagenomics. PLOS ONE. 2012 Feb 3;7(2):e30126.

16. Morgan M. DirichletMultinomial: Dirichlet-Multinomial Mixture Model Machine Learning for Microbiome Data. 2019.

17. Lê S, Josse J, Husson F. FactoMineR: A Package for Multivariate Analysis. J Stat Softw. 2008;25(1):1–18.

18. Fortin JP, Labbe A, Lemire M, Zanke BW, Hudson TJ, Fertig EJ, et al. Functional normalization of 450k methylation array data improves replication in large cancer studies. Genome Biol. 2014;15(12):503.

19. Yan Q, Forno E, C. Celedón J, Chen W. A region-based method for causal mediation analysis of DNA methylation data. Epigenetics. 2022 Mar 4;17(3):286–96.

20. Gomez L, Odom GJ, Young JI, Martin ER, Liu L, Chen X, et al. coMethDMR: accurate identification of co-methylated and differentially methylated regions in epigenome-wide association studies with continuous phenotypes. Nucleic Acids Res. 2019 Sep 26;47(17):e98–e98.

21. Brooks ME, Kristensen K, Benthem KJ van, Magnusson A, Berg CW, Nielsen A, et al. glmmTMB Balances Speed and Flexibility Among Packages for Zero-inflated Generalized Linear Mixed Modeling. R J. 2017;9(2):378–400.

22. Bates D, Mächler M, Bolker B, Walker S. Fitting Linear Mixed-Effects Models Using lme4. J Stat Softw. 2015 Oct 7;67:1–48.

23. Geissinger EA, Khoo CLL, Richmond IC, Faulkner SJM, Schneider DC. A case for beta regression in the natural sciences. Ecosphere. 2022;13(2):e3940.

24. Douma JC, Weedon JT. Analysing continuous proportions in ecology and evolution: A practical introduction to beta and Dirichlet regression. Methods Ecol Evol. 2019;10(9):1412–30.

25. Villicaña S, Bell JT. Genetic impacts on DNA methylation: research findings and future perspectives. Genome Biol. 2021 Apr 30;22(1):127.

26. Zeilinger S, Kühnel B, Klopp N, Baurecht H, Kleinschmidt A, Gieger C, et al. Tobacco Smoking Leads to Extensive Genome-Wide Changes in DNA Methylation. PLOS ONE. 2013 May 17;8(5):e63812.

27. Horvath S, Raj K. DNA methylation-based biomarkers and the epigenetic clock theory of ageing. Nat Rev Genet. 2018 Jun;19(6):371–84.

28. Grant OA, Wang Y, Kumari M, Zabet NR, Schalkwyk L. Characterising sex differences of autosomal DNA methylation in whole blood using the Illumina EPIC array. Clin Epigenetics. 2022 May 14;14(1):62.

29. Stekhoven DJ, Bühlmann P. MissForest—non-parametric missing value imputation for mixed-type data. Bioinformatics. 2012 Jan 1;28(1):112–8.

30. Hartig F, Lohse L. DHARMa: Residual Diagnostics for Hierarchical (Multi-Level / Mixed) Regression Models [Internet]. 2022 [cited 2022 Nov 2]. Available from: https://CRAN.R-project.org/package=DHARMa

31. Maksimovic J, Oshlack A, Phipson B. Gene set enrichment analysis for genome-wide DNA methylation data. Genome Biol. 2021 Jun 8;22(1):173.

32. Alexa A, Rahnenführer J. Gene set enrichment analysis with topGO [Internet]. Available; 2009 [cited 2017 Apr 6]. Available from: https://bioconductor.riken.jp/packages/3.2/bioc/vignettes/topGO/inst/doc/topGO.pdf

33. Manjang K, Tripathi S, Yli-Harja O, Dehmer M, Emmert-Streib F. Graph-based exploitation of gene ontology using GOxploreR for scrutinizing biological significance. Sci Rep. 2020 Oct 7;10(1):16672.

34. Yu G, Li F, Qin Y, Bo X, Wu Y, Wang S. GOSemSim: an R package for measuring semantic similarity among GO terms and gene products. Bioinformatics. 2010 Apr 1;26(7):976–8.

35. Bakdash JZ, Marusich LR. Repeated Measures Correlation. Front Psychol [Internet]. 2017 [cited 2023 Jun 2];8. Available from: https://www.frontiersin.org/articles/10.3389/fpsyg.2017.00456

36. Bakdash JZ, Marusich LR. Corrigendum: Repeated Measures Correlation. Front Psychol [Internet]. 2019 [cited 2023 Jun 2];10. Available from: https://www.frontiersin.org/articles/10.3389/fpsyg.2019.01201

37. Alexa A, Rahnenfuhrer J, Lengauer T. Improved scoring of functional groups from gene expression data by decorrelating GO graph structure. Bioinformatics. 2006 Jul 1;22(13):1600–7.

38. Alexa A, Rahnenfuhrer J. topGO: Enrichment Analysis for Gene Ontology. R package version 2.38.1 [Internet]. 2019. Available from: https://bioconductor.org/packages/release/bioc/html/topGO.html

39. O'Leary NA, Wright MW, Brister JR, Ciufo S, Haddad D, McVeigh R, et al. Reference sequence (RefSeq) database at NCBI: current status, taxonomic expansion, and functional annotation. Nucleic Acids Res. 2016 Jan 4;44(D1):D733-745.

40. Landrum MJ, Lee JM, Benson M, Brown GR, Chao C, Chitipiralla S, et al. ClinVar: improving access to variant interpretations and supporting evidence. Nucleic Acids Res. 2018 Jan 4;46(D1):D1062–7.

41. Amberger JS, Bocchini CA, Scott AF, Hamosh A. OMIM.org: leveraging knowledge across phenotype–gene relationships. Nucleic Acids Res. 2019 Jan 8;47(D1):D1038–43.

42. Gandal MJ, Haney JR, Parikshak NN, Leppa V, Ramaswami G, Hartl C, et al. Shared molecular neuropathology across major psychiatric disorders parallels polygenic overlap. Science. 2018 Feb 9;359(6376):693–7.

43. Hothorn T, Buehlmann P, Kneib T, Schmid M, Hofner B, Otto-Sobotka F, et al. mboost: Model-Based Boosting [Internet]. 2021 [cited 2021 May 5]. Available from: https://CRAN.R-project.org/package=mboost

44. Leek JT, Storey JD. Capturing Heterogeneity in Gene Expression Studies by Surrogate Variable Analysis. PLOS Genet. 2007 Sep 28;3(9):e161.

45. Leek JT, Johnson W, Parker H, Fertig EJ, Jaffe AE, Zhang Y, et al. sva: Surrogate Variable Analysis [Internet]. Available from: https://www.bioconductor.org/packages/release/bioc/html/sva.html

46. Meinshausen N, Bühlmann P. Stability selection. J R Stat Soc Ser B Stat Methodol. 2010;72(4):417–73.

47. Hofner B, Boccuto L, Göker M. Controlling false discoveries in high-dimensional situations: boosting with stability selection. BMC Bioinformatics. 2015 May 6;16(1):144.

48. Chicco D, Jurman G. The Matthews correlation coefficient (MCC) should replace the ROC AUC as the standard metric for assessing binary classification. BioData Min. 2023 Feb 17;16(1):4.

49. Chicco D, Tötsch N, Jurman G. The Matthews correlation coefficient (MCC) is more reliable than balanced accuracy, bookmaker informedness, and markedness in two-class confusion matrix evaluation. BioData Min. 2021 Feb 4;14(1):13.

50. Chang CC, Chow CC, Tellier LC, Vattikuti S, Purcell SM, Lee JJ. Second-generation PLINK: rising to the challenge of larger and richer datasets. GigaScience. 2015 Dec 1;4(1):s13742-015-0047–8.

51. Purcell S. PLINK v1.90b7 64-bit [Internet]. 2023. Available from: http://pngu.mgh.harvard.edu/purcell/plink/

52. Das S, Forer L, Schönherr S, Sidore C, Locke AE, Kwong A, et al. Next-generation genotype imputation service and methods. Nat Genet. 2016 Oct;48(10):1284–7.

53. Loh PR, Danecek P, Palamara PF, Fuchsberger C, A Reshef Y, K Finucane H, et al. Reference-based phasing using the Haplotype Reference Consortium panel. Nat Genet. 2016 Nov;48(11):1443–8.

54. Clayton D. snpStats: SnpMatrix and XSnpMatrix classes and methods [Internet]. Bioconductor version: Release (3.17); 2023 [cited 2023 Jul 26]. Available from: https://bioconductor.org/packages/snpStats/

55. Pinheiro J, Bates D, DebRoy S, Sarkar D, R Core Team. nlme: Linear and Nonlinear Mixed Effects Models [Internet]. 2019. Available from: https://CRAN.R-project.org/package=nlme

56. Myers TA, Chanock SJ, Machiela MJ. LDlinkR: An R Package for Rapidly Calculating Linkage Disequilibrium Statistics in Diverse Populations. Front Genet [Internet]. 2020 [cited 2023 Sep 25];11. Available from: https://www.frontiersin.org/articles/10.3389/fgene.2020.00157

57. Machiela MJ, Chanock SJ. LDlink: a web-based application for exploring population-specific haplotype structure and linking correlated alleles of possible functional variants. Bioinformatics. 2015 Nov 1;31(21):3555–7.

58. Smigielski EM, Sirotkin K, Ward M, Sherry ST. dbSNP: a database of single nucleotide polymorphisms. Nucleic Acids Res. 2000 Jan 1;28(1):352–5.

59. Sollis E, Mosaku A, Abid A, Buniello A, Cerezo M, Gil L, et al. The NHGRI-EBI GWAS Catalog: knowledgebase and deposition resource. Nucleic Acids Res. 2023 Jan 6;51(D1):D977–85.

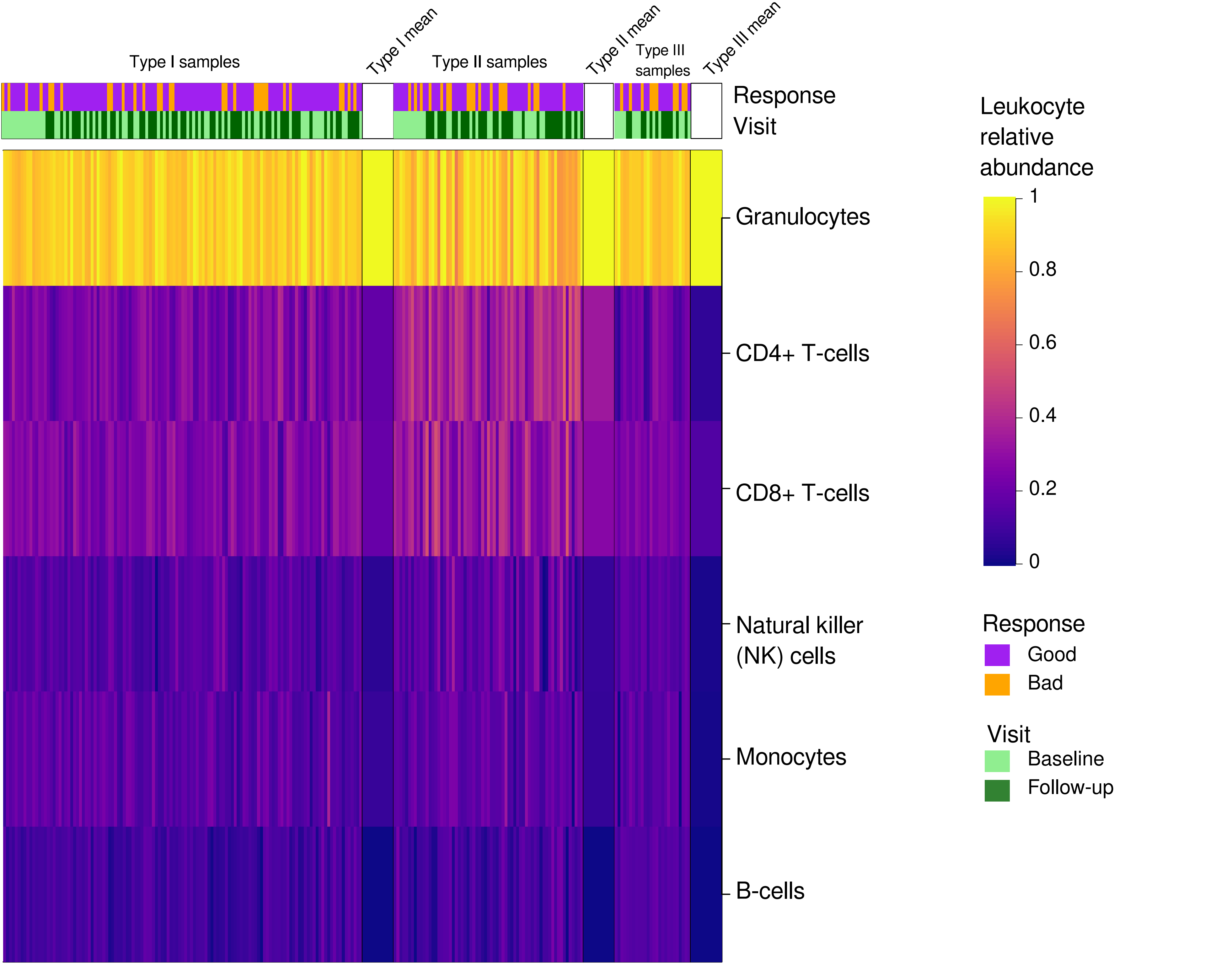


**Figure S1**. Variability of leukocyte composition estimated based on the methylation data. The heatmap shows relative abundances of six leukocyte types in the individual samples (narrow columns) and their mean values for the three “leukocyte community” types (“Dirichlet components”, wide columns) identified by Dirichlet multinomial mixture modelling. All three types are characterized by high proportion of granulocytes (neutrophils) and they differ mainly by the relative abundances of CD4+ and CD8+ T-cells. Sample annotation (response and visit) is shown on the top of the figure. There is no significant correlation between the lekocyte community type and the response to amisulpride, despite the significant difference in the relative abundance of natural killer cells in the univariate tests (Table S1). The samples are ordered according to the default parameters of the plotting function (*heatmapdmn* in the R *DirichletMultinomial* package).

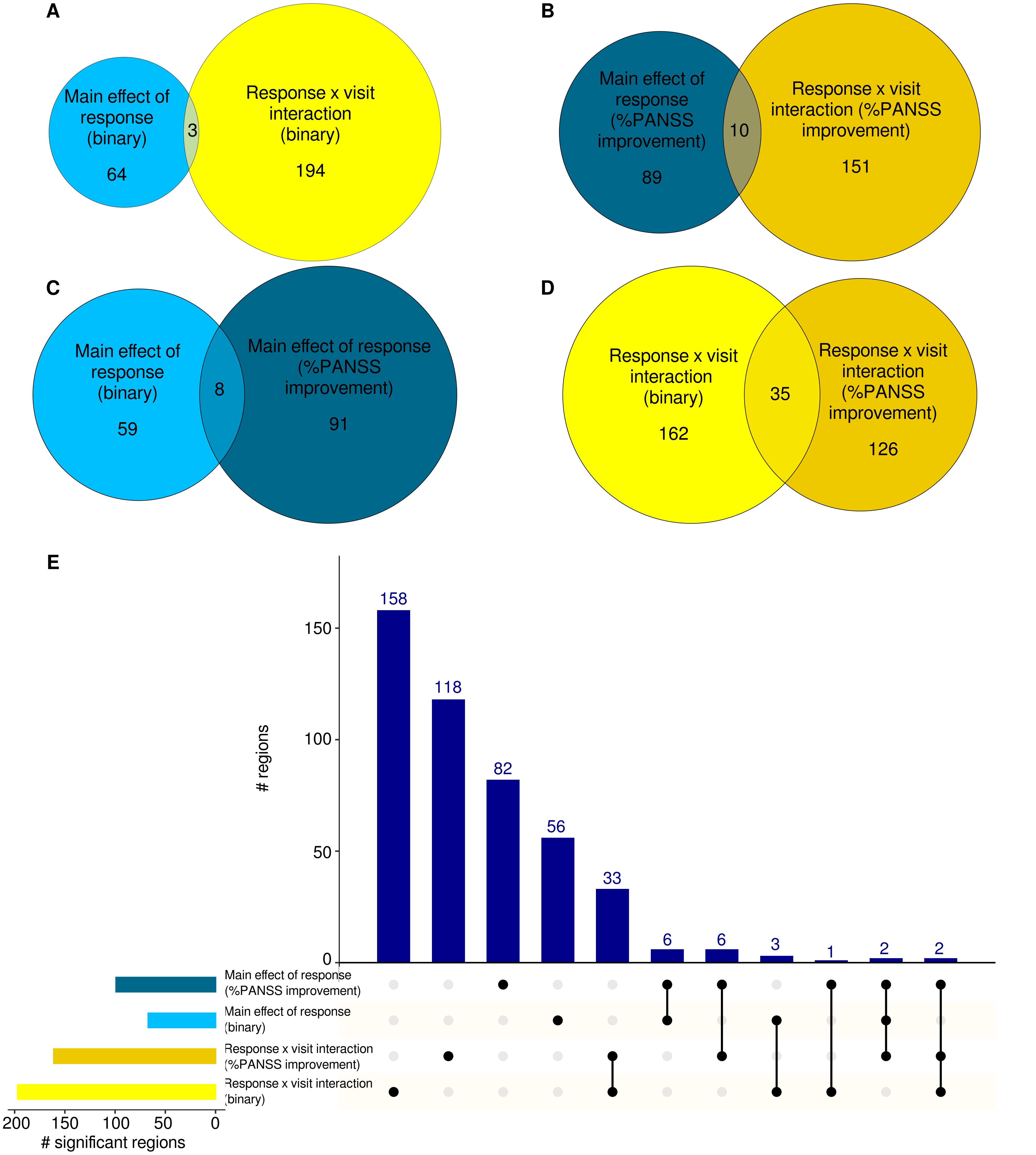


**Figure S2**. Overlap between the groups of regions whose methylation is significantly associated with response to amisulpride according to different criteria. The first row shows overlap between the regions with average methylation differences between the good and bad responders ("main effect of response) and those with response-specific treatment effect ("response x visit interaction") for the response defined as (A) dichotomous (yes/no) or (B) continuous (%PANSS improvement) variable. Panel (C) shows overlap of the regions significant for the main effect of response between the models with different response definitions (binary vs. continuous), panel (D) shows the corresponding overlap for the regions with response-specific treatment effect. Whereas the panels A-D show overlaps between the pairs of the region subsets, (E) is an upset plot showing also three-way intersections (no region was found in all four groups). Colors of the bars showing the total number of significant regions in the (E) correspond to the colors in the panels A-D.

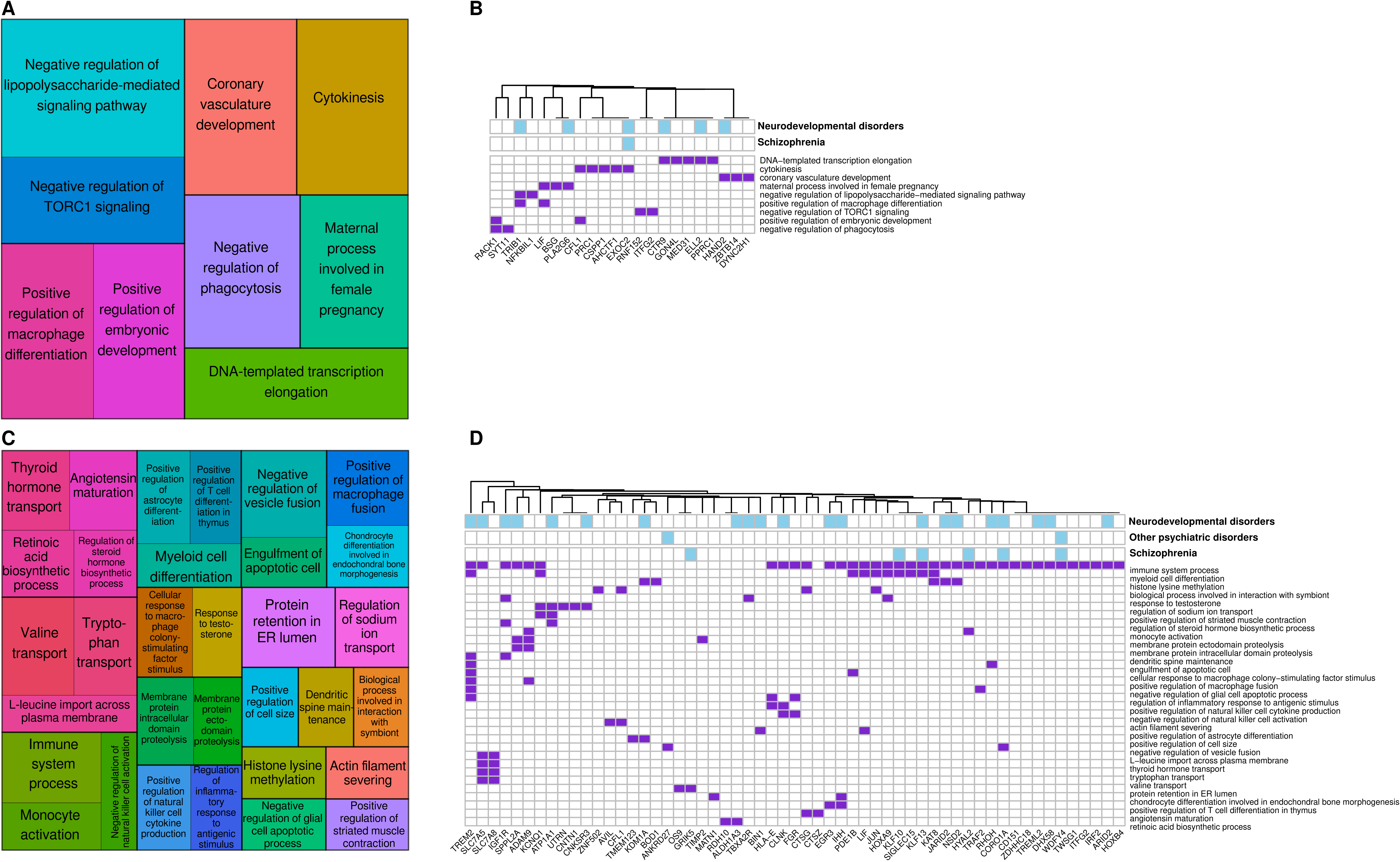


**Figure S3**. Biological processes (BP) enriched in the regions whose methylation co-varies with the %PANSS improvement (A) and in those with response-specific treatment effect (C), with the heatmaps showing the differentially methylated genes within the respective enriched terms (B, D). Terms in A & C were filtered to reduce redundancy and grouped by semantic similarity, with similar terms represented by the shades of the same color, and the rectangle size is inversely proportional to the corresponding term p-value. Genes annotation at the top of the heatmaps (B & D) show if the gene has previously been associated with schizophrenia, other psychiatric disorders and eurodevelopmental and neurodegenerative conditions based on the ClinVar, OMIM, MedGen and GWAS catalog databases.

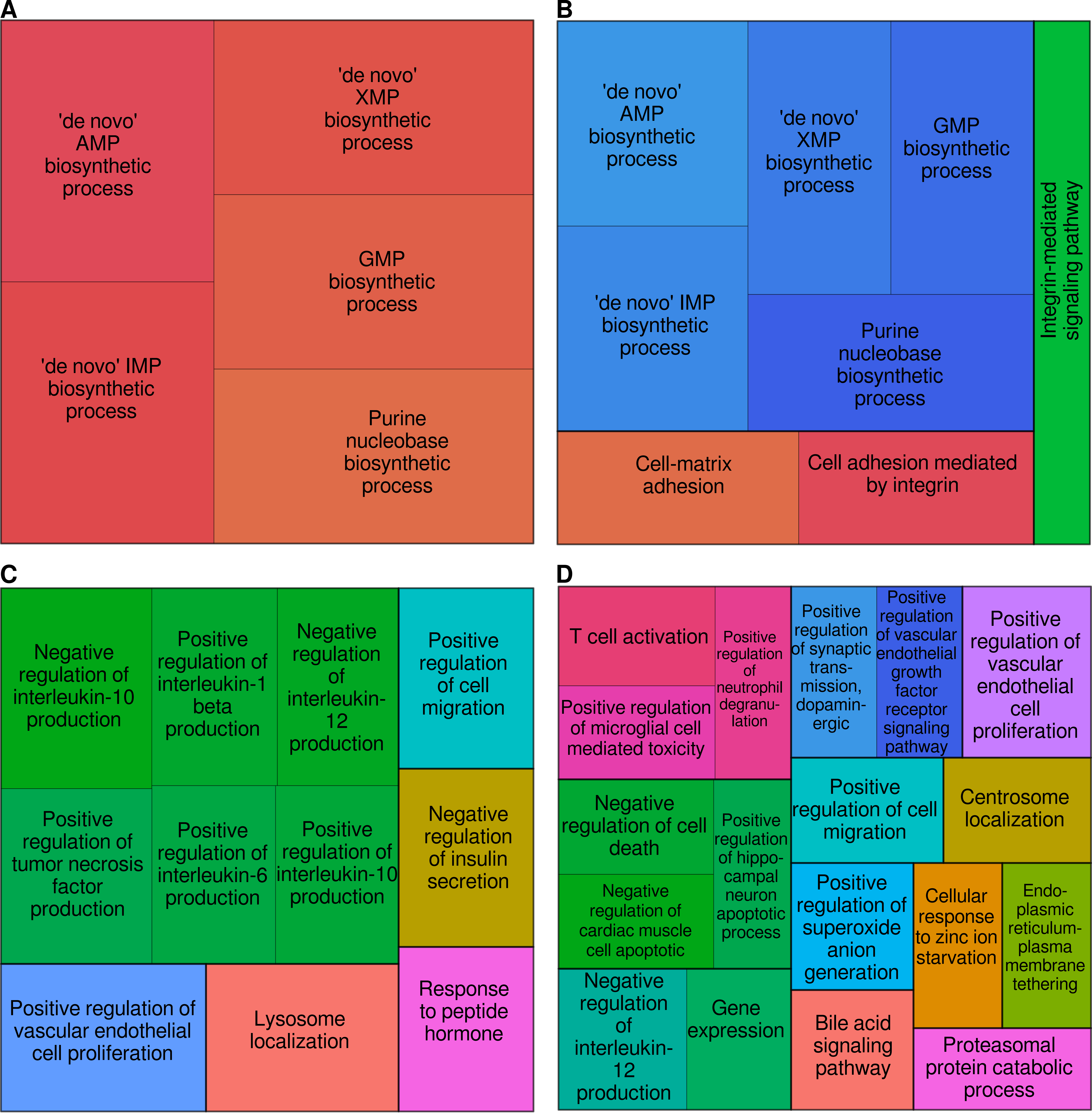


**Figure S4.** Biological processes (BP) enriched in the genes whose expression is correlated with the methylation of the regions whose methylation differs on average between the good and bad responders (A-B) or those with response-specific treatment effect (C-D). A & C show the results for the genes whose expression is correlated with the methylation of individual CpGs within the regions of interest, B & D show the enrichment results for the genes with expression correlating with the whole-region methylation. The results are based on topGO weight01 algorithm. Terms were filtered to reduce redundancy and grouped by semantic similarity. Rectangle size is inversely proportional to a term's p-value.

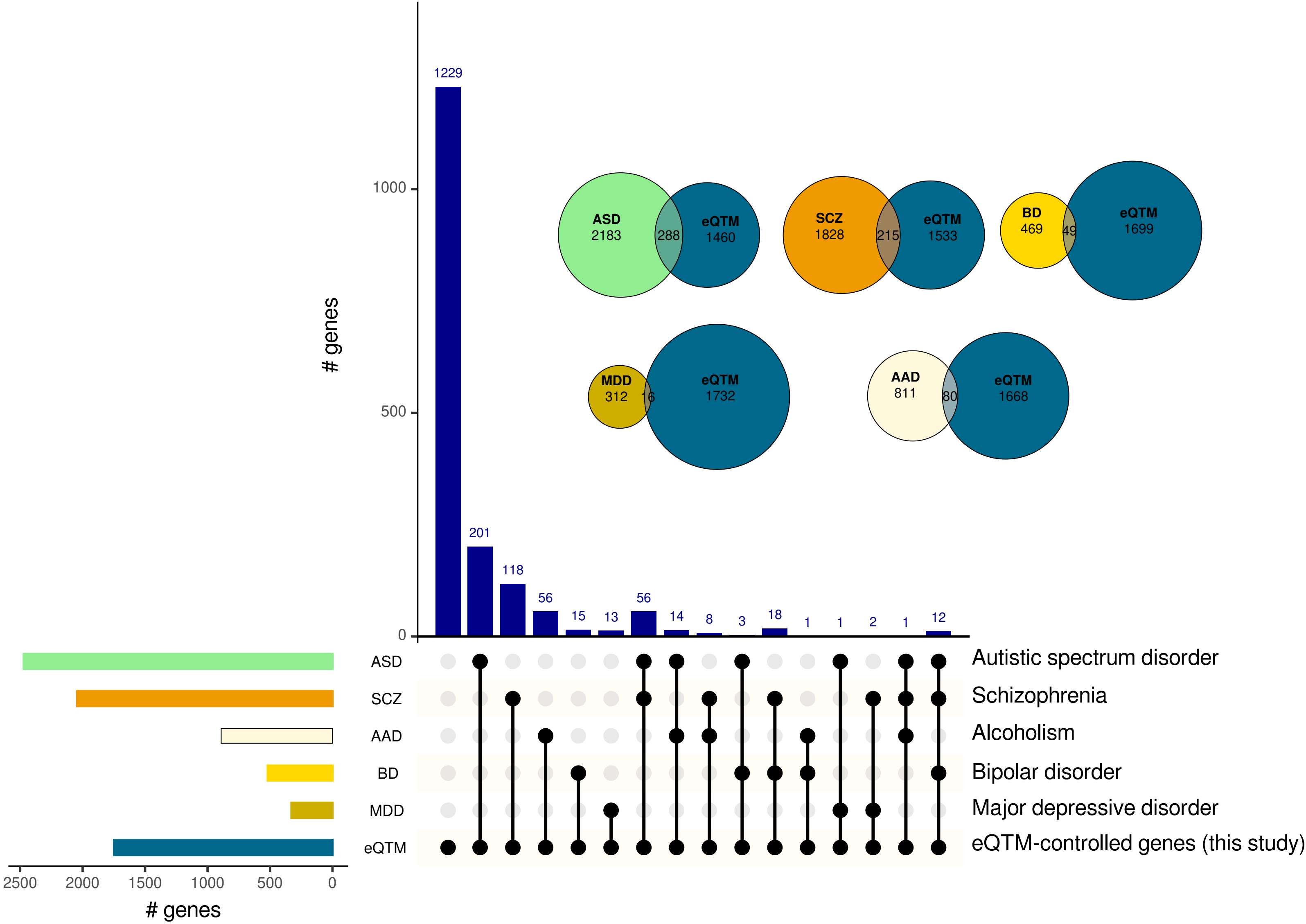


**Figure S5**. Overlap between the genes identified by expression quantitative trait methylation analysis (eQTM) and whose expression is thus correlated with the methylation of response-associated regions, and the genes dysregulated in several psychiatric disorders according to Gandal et al., *Science* 2018. The upset plot shows all the intersections with eQTM-identified genes, Venn diagrams in the inset show pairwise overlap of the eQTM-identified genes with each of the psychiatric disorders separately.

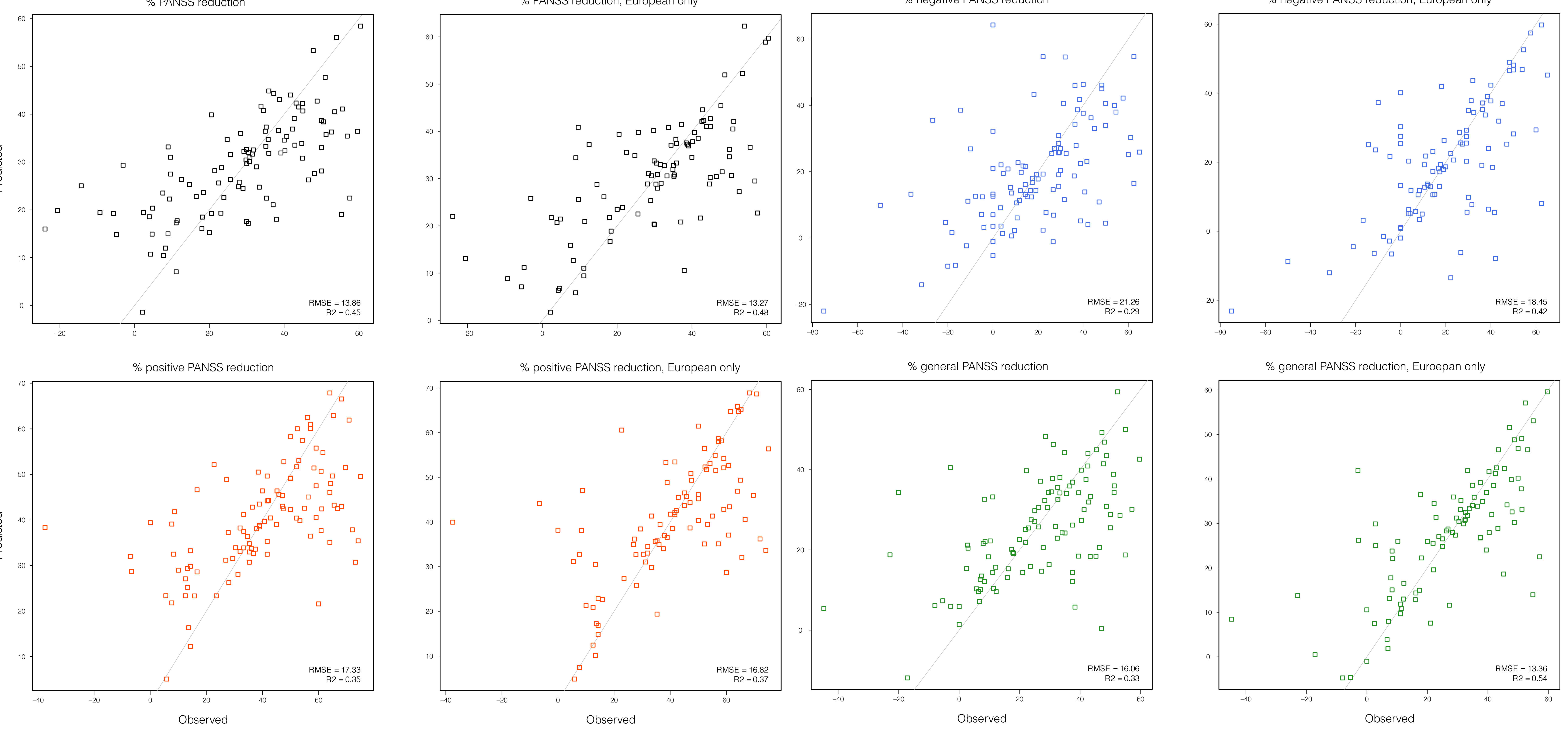


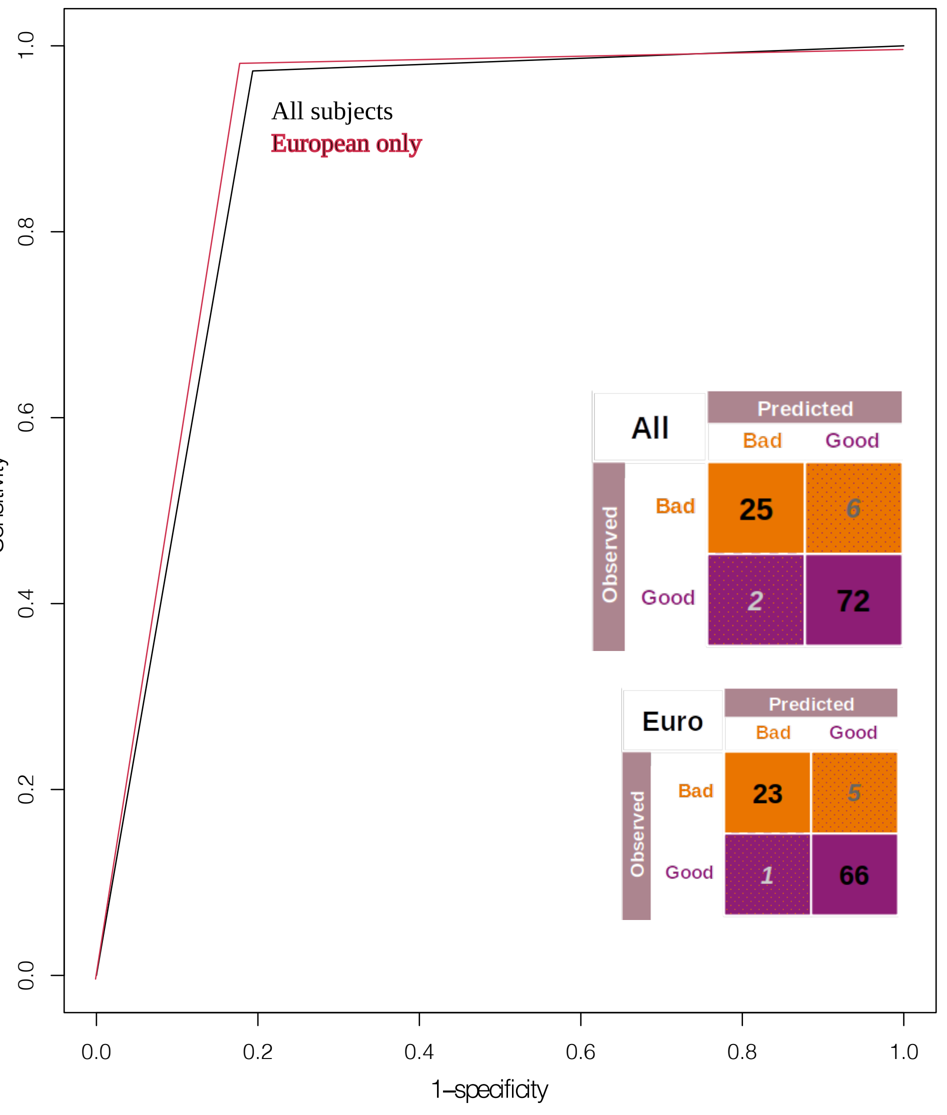


**Figure S6**. Performance of predictive models. The first two rows show plots of observed vs predicted values for total, negative (first row), positive and general (second row) PANSS reduction, with models based on all subjects on the left and Europeans only on the right. Receiver operator characteristic (ROC) curves for all (black) and European only (red) subjects are shown in third row, with the corresponding confusion matrices in the inset.

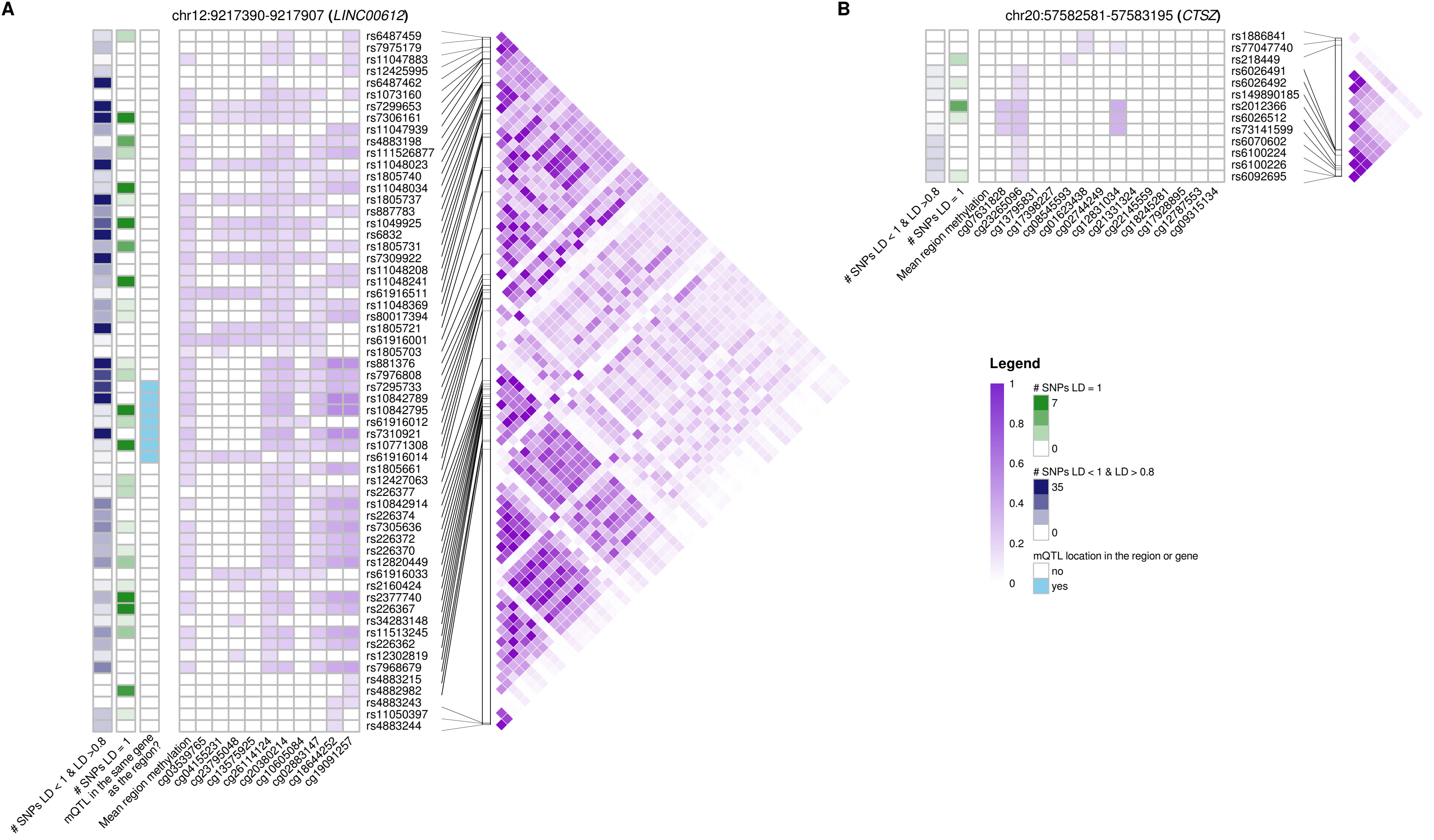


**Figure S7**. Association ($R^2$) between the methylation in the regions predicting %PANSS improvement and their corresponding mQTLs: (A) *LINC00612* and (B) *CTSZ*. The association was calculated for each CpG in a region separately and for the mean region methylation value. LD = 1 shows the number of SNPs in complete linkage for each mQTL, LD > 1 shows the number of linked SNPs (LD > 0.8) excluding those in complete linkage. "mQTL in the region" shows if a particular SNP is located in the response-related co-methylated region, "mQTL in the same gene as the region?" marks the SNPs that are annotated to the same gene as the corresponding co-methylated region. Counts in annotation columns are capped at 7 and 35 (based on 75% quantile) for clarity. Genomic coordinates are based on GRCh37. SNP rs6313 in (B) is shown in bold.

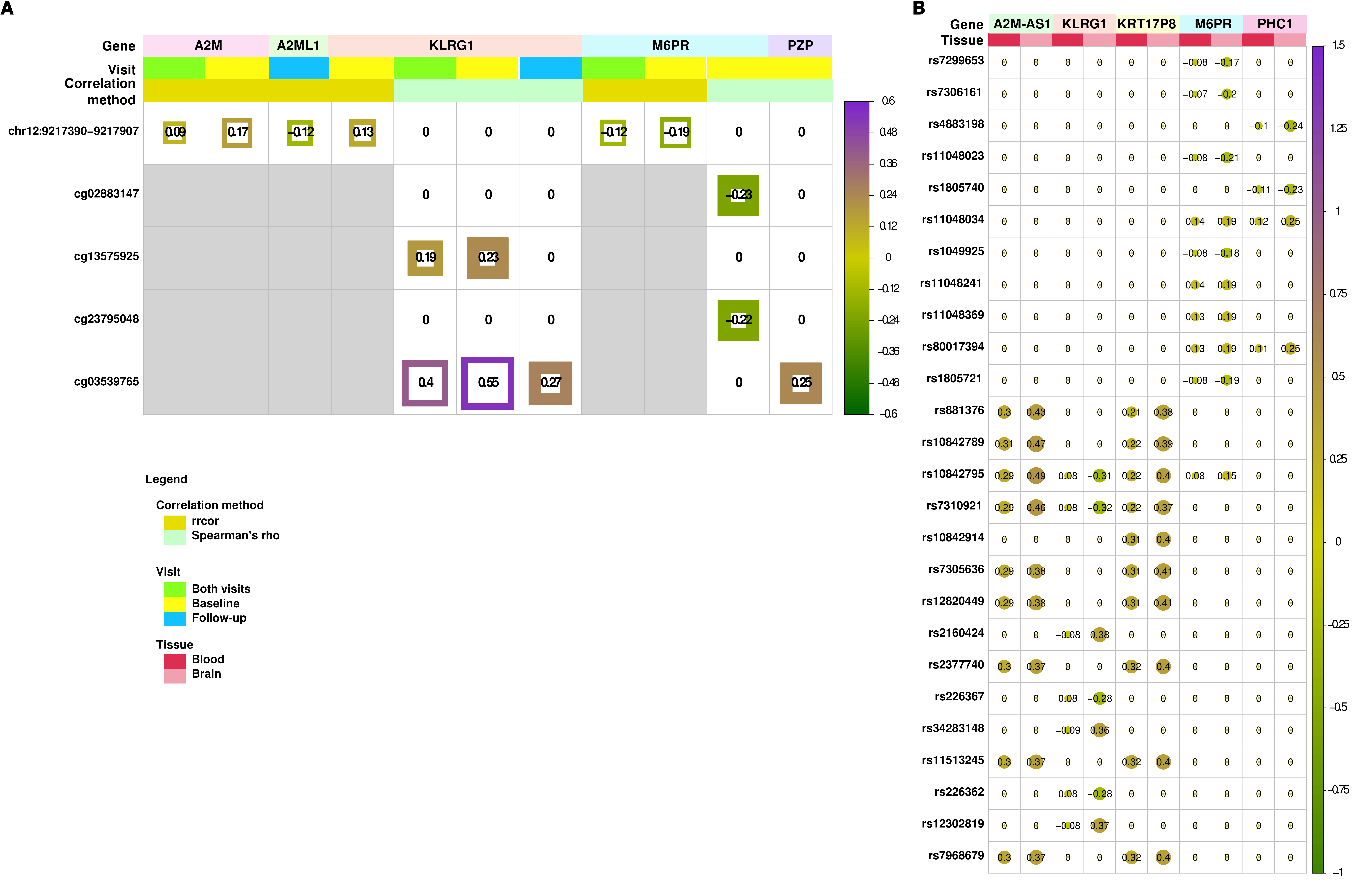


**Figure S8**. Correlations detected between the gene expression and methylation of the PANSS reduction predicting region LINC00612 in this study (A) and the effect of the mQTLs associated with this region on the gene expression in the blood and brain tissues according to GTEX8 database (B). Correlations were calculated for the mean methylation value of the region (Spearman's rho), for the whole region accounting for the variability of individual CpGs within the region (rrcor) and for the individual CpGs in the region (Spearman's rho), for both visits together and for each visit separately. Rectangle size is proportional to the strength of correlation, the thickness is proportional to the width of confidence intervals. For (B), association with the gene expression is shown only if the SNP affects the expression of the gene in both blood and brain. The effect sizes are slopes of the linear regression, computed as the effect of the alternative allele relative to the reference one according to the GTEX portal FAQ. The size of the circle is proportional to the effect size.

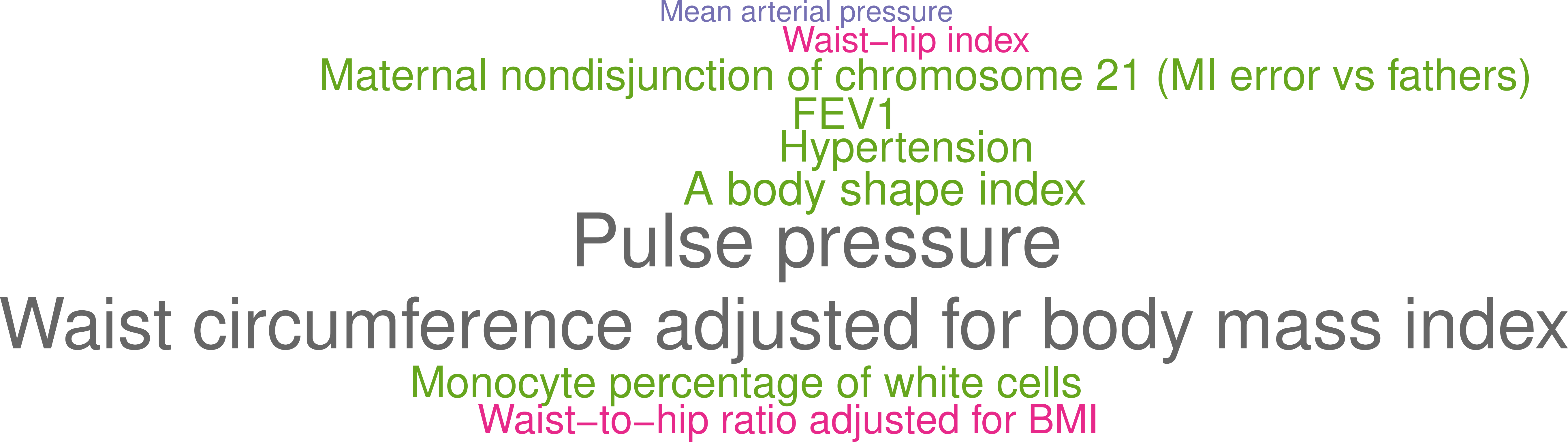


**Figure S9**. Wordcloud of over-represented (raw Fisher's exact p-value <= 0.05) GWAS traits associated with the SNPs that act as mQTLs for the HOXA and HTR2A co-methylated regions. Over-representation of the GWAS traits associated with the mQTLs was calculated relative to the traits associated with the set containing all SNPs tested for the link with methylation. SNPs that are in the linkage ($R^2 > 0.8$) with the tested SNPs were also included. The colors are chosen by the software randomly and have no meaning except to increase readability. The font size is proportional to the frequency of the trait in the gene set.

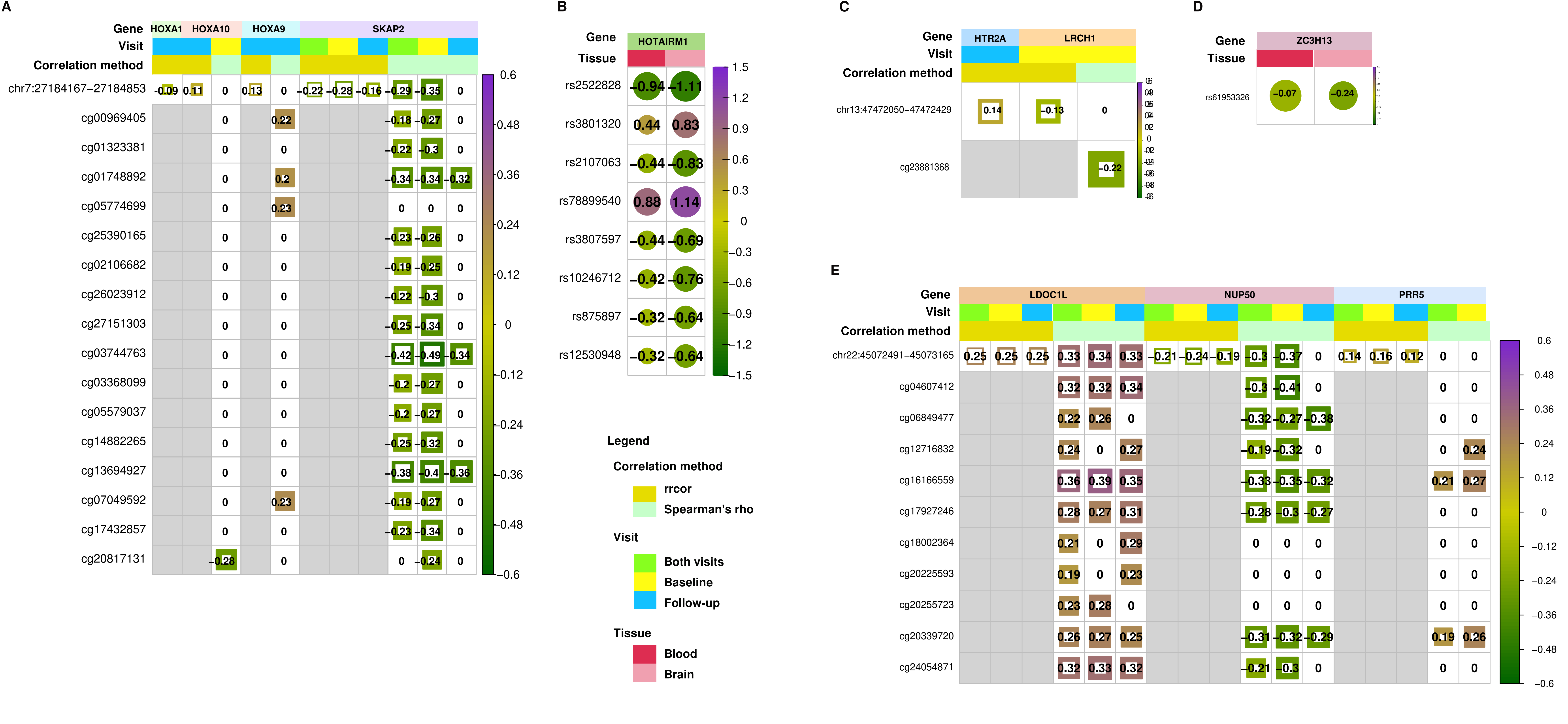


**Figure S10**. Correlations between the gene expression and methylation of the binary response (yes/no) predicting regions detected in this study: A) HOXA, C) HTR2A and E) PRR5, and the effect of the mQTLs associated with these regions on the gene expression in the blood and brain tissues according to GTEX8 database: B) HOXA, D) HTR2A. Correlations were calculated for the mean methylation value of the regions (Spearman's rho), for the whole regions accounting for the variability of individual CpGs within the regions (rrcor) and for the individual CpGs in the regions (Spearman's rho), for both visits together and for each visit separately. Rectangle size in A, C & E is proportional to the strength of correlation, the thickness is proportional to the width of confidence intervals. For B & D, association with the gene expression is shown only if the SNP affects the expression of the gene in both blood and brain. The effect sizes are slopes of the linear regression, computed as the effect of the alternative allele relative to the reference one according to the GTEX portal FAQ. The size of the circle is proportional to the effect size.
